# An agent-based learning model integrating sex differences in renal cell carcinoma

**DOI:** 10.3389/fimmu.2026.1779638

**Published:** 2026-04-01

**Authors:** Emanuela Merelli, Tarek Taha, Marco Caputo, Destiny Obude, Edoardo Papa, Alessandro Rizzo, Sebastiano Buti, Francesco Massari, Javier Molina-Cerrillo, Fernando Sabino Marques Monteiro, Brigida Anna Maiorano, Matteo Santoni

**Affiliations:** 1 School of Sciences and Technology, University of Camerino, Camerino, MC, Italy; 2 Royal Marsden NHS Foundation Trust, London, United Kingdom; 3 Istituto di Ricovero e Cura a Carattere Scientifico (IRCCS) Istituto Tumori “Giovanni Paolo II”, Bari, Italy; 4 Medical Oncology Unit, University Hospital of Parma, Parma, Italy; 5 Department of Medicine and Surgery, University of Parma, Parma, Italy; 6 Medical Oncology, Istituto di Ricovero e Cura a Carattere Scientifico (IRCCS) Azienda Ospedaliero-Universitaria di Bologna, Bologna, Italy; 7 Department of Medical and Surgical Sciences (DIMEC), University of Bologna, Bologna, Italy; 8 Department of Medical Oncology, Hospital Ramón y Cajal, Madrid, Spain; 9 ARON Research Foundation Ente del Terzo Settore (ETS), Macerata, Italy; 10 Latin American Cooperative Oncology Group - LACOG, Porto Alegre, Brazil; 11 Oncology and Hematology Department, Hospital Sírio Libanês, Brasília, Brazil; 12 Department of Medical Oncology, Istituto di Ricovero e Cura a Carattere Scientifico (IRCCS) San Raffaele Hospital, Milan, Italy; 13 Medical Oncology Unit, Macerata Hospital, Macerata, Italy

**Keywords:** Agent-Based Model (ABM), Agent-Learning Model (ALM), computational simulation, immune system response, immunotherapy, machine learning, RCC

## Abstract

**Background:**

Sex-based differences influence tumor biology, immune responses, and treatment outcomes in renal cell carcinoma (RCC), yet most computational models do not jointly incorporate sex hormones, immune composition, and tumor genetic evolution. Agent-based models (ABMs) effectively simulate tumor–immune interactions but are rarely extended to include sex-specific modulation or machine learning–based optimization. This study enhanced an agent-based learning model (ALM) to simulate RCC progression and treatment response by integrating hormonal effects, immune interactions, and tumor genetic adaptation with data-driven tuning.

**Methods:**

An RCC-specific ALM was developed incorporating immune agents (CD8+, NK, Treg, dendritic cells), hormone-sensitive mechanisms, tumor genetic modules, and effects of immune checkpoint inhibitors (*ICI*s) and tyrosine kinase inhibitors (*TKI*s). Tumor evolution was modeled using a genetic algorithm simulating promoter and gene mutations, with fitness defined by immune evasion and proliferation advantages. Model parameters were optimized using clinical outcomes from the ARON dataset via the Optuna framework, and performance was assessed using concordance index (*CI*) and mean squared error (*MSE*).

**Results:**

Simulations reproduced sex-specific treatment responses. Female models showed delayed initial responses but stronger late immune activation and rapid tumor regression, whereas male models exhibited more stable early responses but greater tumor resilience driven by genetic adaptations. Adaptive learning showed capability of reducing prediction error with both fitness functions.

**Conclusions:**

This ALM offers an exploratory framework to provide preliminary insights into how sex hormones, immune dynamics, and tumor genetics may jointly contribute to shaping RCC treatment outcomes. Although the limited sample size constrains validation, the results suggest the potential of combining ABMs with biological data-driven optimization to support patient prediction and call for further investigation in larger cohorts.

## 1 Introduction

Sex-based differences critically influence tumor biology, antitumor immunity, treatment-related toxicities, and therapeutic outcomes, especially in the context of immunotherapy (1, 2). Renal cell carcinoma (RCC) exemplifies an immune-responsive cancer with significant variability in patient outcomes, reflecting the interaction of tumor genetics, immune editing, and the modulatory effects of sex hormones. However, sex-hormone–mediated immune modulation alone does not account for this heterogeneity. Tumor evolution is also driven by mechanisms such as antigenic variation, immune suppression, and strategies that enable evasion, which undermine both innate and adaptive immunity (3, 4). Additional factors like comorbidities and metabolic state may also influence immune responsiveness (5).

Hormonal regulation is a key factor, alongside tumor-intrinsic genetics and immune-escape mechanisms. Estrogen has been reported to modulate antitumor immunity by influencing the activity of T cells, B cells, natural killer (NK) cells, macrophages, and dendritic cells, as well as enhancing the production of pro-inflammatory cytokines (e.g., interleukin [IL]-1, IL-6, tumor necrosis factor [TNF]-α) and regulating immune checkpoint molecules like Programmed death (PD)-1 and PD-Ligand (PD-L)1 (6, 7). These effects may lead to increased responsiveness to immune checkpoint inhibitors (*ICI*s) but also carry a higher risk of autoimmune manifestations.

Conversely, testosterone reduces T and NK cell activity, lowers cytokine secretion, and downregulates immune-related gene expression, thereby fostering an immunosuppressive environment that can support tumor growth (6, 8, 9). Epidemiological and experimental studies in RCC suggest that androgen receptor signaling may increase tumor aggressiveness, while estrogen receptor pathways are linked to antiproliferative and immune-modulatory effects (10–12). Current biomarkers used to predict responses in RCC, such as PD-L1 expression and tumor mutational burden (TMB), only capture parts of this complexity, highlighting the need for computational frameworks that integrate genetic, immunological, and hormonal factors to better forecast *ICI* response and resistance.

Agent-based models (ABMs) offer a framework for studying tumor–immune interactions (13–16). Early models viewed immune cells as uniform populations driven by ordinary differential equations (17), while newer methods include spatial heterogeneity, immune editing, and escape mechanisms (18–21). Sex hormones have been modeled in infections, showing testosterone’s role in suppressing T-cell activity, but ABMs specific to RCC are limited. Adipose cytokine–tumor interactions have been used to explore “the obesity paradox” in RCC (14), although they did not account for sex differences or adaptive learning. Incorporating sex-specific immunobiology into computational models could uncover new therapeutic targets and improve trial stratification. To fill this gap, we developed an RCC-specific ABM that integrates sex hormone effects, tumor genetics, PD-1/PD-L1 dynamics, and an adaptive learning component inspired by approximate Bayesian computation (ABC) (22).

## 2 Materials and methods

### 2.1 Biological function

The main biological function modeled by our system describes the immune response to immunotherapy in RCC, accounting for patient-specific differences in sex and immune profile by extending our previous work regarding an agent-based model for reproducing the immune response for the obesity paradox in RCC (14). We define an observed function of the form.


$$f(Sex,\bar{X})=(OS,Survived)$$

where

Sex∈{Female. Male} is the biological sex of the patient;

$\bar{X}=[N_{CD8+},N_{NK},N_{Treg}\ldots N_{TCellHZ},BMI]$ is the vector composed of immunological and physiological variables, as the number of various immune cells and body mass index (BMI);

$OS\in {\mathbb{R}}_{\geq 0}\cup \;\{\infty \}$ is the total survival time, that is, the observed or predicted survival time, representing an aggregate clinical outcome;

Survived ∈{true, false} is the binary output that represents whether the patient survives during the simulated time course.

The selection of the parameters denoted by the vector *X* is based on known biological factors that influence the success of immunotherapy in RCC. For instance, CD8+ T cells and NK cells are crucial in directly destroying tumor cells, while regulatory T cells (Tregs) are known to suppress immune responses and may promote tumor growth. Including these cell types enables the model to reflect the intricate balance between anti-tumor and pro-tumor immune forces.

BMI is also included due to its known impact on immune regulation and therapy effectiveness. Extreme BMI values can alter the balance of immune cell types and hormone levels, indirectly influencing how well immunotherapy works.

Patient sex is included as a key variable *Sex*, because hormonal differences between male and female patients, such as variations in estrogens or testosterone, can affect immune behavior at multiple levels, influencing both cell activation and apoptosis rates. We are aware that the influence of sex hormones is subject to the patients’ age, however, in this first stage, we leave the study as future work.

The function *f* includes the dynamic interaction between the immune system and the tumor, as well as external regulators like hormones that vary depending on sex and BMI. Both aspects are crucial for realistically capturing how the body responds to cancer. Specifically, we simulate the hormone-sensitive processes that regulate CD8+ T-cell activation and proliferation, cytotoxic T-cell apoptosis and killing efficiency, as well as antigen presentation and recognition thresholds. These mechanisms are mathematically represented through parameter updates that respond to local hormone sensing (such as estrogen and testosterone), which are modeled at the agent level.

The output tuple (OS, Survived) thus captures the effectiveness of the immune response in the simulation, including both biological variability and random cell-level interactions. Overall, this function lets us measure how variables like sex and BMI influence patient outcomes under immunotherapy, supporting comparative simulations and the generation of biologically motivated hypotheses.

### 2.2 Tumor environment

Our environment includes blood vessels that carry oxygen and hormones, and we also depict angiogenesis, which promotes tumor spread. For a more comprehensive description of each component of the environment refer to **Supplementary Information** in which **Supplementary Figure 1** provides a stylized version of the user interface of the simulated environment. The initial configuration of the environment for the simulation is influenced by BMI and sex. Regarding BMI, obesity influences tumor formation by altering parameter ranges, the number of adipocytes, and dynamic properties. Gender impacts the hormonal profile, changes in cell concentration, and the initial mutation load. Since hormone levels depend directly on the patient’s sex, adjustments can be made in the initialization of cancer therapy to evaluate how different drugs affect patients of different genders.

### 2.3 Cell interaction paradigm

The communication among cells relies on the *interaction-as-perception* paradigm proposed in our previous work (23). Each agent is endowed with a search dimension $d_{s}$, representing the extent of the surrounding space it is perceived. When the Euclidean distance between two agents falls below $d_{s}$, a directed edge is added to a transient interaction graph ${\text{G}}_{t}$ for that time step *t*. Edges trigger state-dependent rules such as cytotoxic attack, antigen presentation, or checkpoint signaling. Reducing $d_{s}$ mimics poor tissue perfusion or stromal barrier, whereas larger values emulate lymphocyte infiltration after vascular-normalizing therapy.

The interactions among its agents shape the model’s dynamics. We can identify two primary types of interactions:

*Cooperation*: Agents belonging to the immune system work together to initiate and sustain the immune response, ensuring stable environmental conditions are maintained or restored. An example of this interaction is that between DCs and CD8+ cells. The DCs capture antigens through phagocytosis and then present them to the CD8+ cells, which become activated and carry out their cytotoxic functions to neutralize the threat. This process occurs in a probabilistic manner. The DC has a state (phagocytized) that influences its actions. When this state is false, the DC approaches an antigen and attempts to capture it with a certain chance. When the state is true, it moves towards an agent (T Cell) that needs activation, triggering a sequence of events through the activated T Cell. DCs require other agents to eliminate antigens, and other immune cells rely on DCs to target their attack, creating a cooperative interaction.

*Competition*: The interaction between tumor cells and all other cells, especially CD8+ cells, is competitive. The CD8+ cells aim to neutralize the tumor cells, while the tumor cells try to suppress them or at least avoid contact. Tumor cells exhibit behaviors, including mutations, that enhance their survival, allowing them to outlast immune cells. These mutations lead to complex evasive maneuvers and increased suppressive capabilities. A key behavior is higher expression of PD-L1, which binds to PD-1 receptors on T cells, effectively exhausting them.

This behavior is modeled as a weighted chance of T cell inhibition, influenced by genetic mutations. The DNA class calculates mutation scores for mutated genes within a range of *[0,1].* Mutated genes are identified by comparing the tumor gene with the wild type from a lookup table. Genes are considered mutated if their sequences differ from the wild type. Through numerous such mutations, tumor cells can improve their chances of survival.

Other less obvious interaction distinctions include the ways hormones signal to cells, which can be both suppressive and stimulatory depending on the situation. Sex hormones may stimulate when at certain levels become inhibitory beyond that point. For example, with estrogen and pregnancy, estrogen is usually pro-inflammatory in the immune system, meaning T cells become more aggressive; however, in specific cases like late pregnancy, when estrogen and progesterone levels are higher, it adopts an anti-inflammatory role. In the simulation, this is implemented using a threshold-based approach with three levels — low, normal, high — applied to all sex hormones. Each level linearly affects certain traits of different cells, regulating them as needed.

### 2.4 Therapy influence

Two categories of drugs modeled and used in the simulation are *ICI*s and Tyrosine Kinase Inhibitors (*TKI*s). These drugs can be combined into single therapies or treatments, as approved by Regulatory Agencies after clinical trials, and then added to a running simulation starting from a specific step.

In our model, *ICI*s are responsible for:

- Blocking the tumor’s ability to bind to PD-1 on T cells’ surfaces, thus preventing the tumor from exhausting these cells. Specifically, at each simulation step, the drug decreases the probability that each tumor cell with the ability to present PD-1 inhibition—due to mutations—interacts with a T cell.
- Partially reawakening previously exhausted T cells to fight the tumor by restoring the level of exhaustion caused by an internal variable.
- Increasing immune infiltration, which refers to the number of immune cells recruited to the TME in response to signals from other cells, is represented in the simulation by additional agents appearing at the edge of the space.

In the same models, *TKI*s are employed for:

- Directly inhibiting tumor growth by decreasing both tumor proliferation and angiogenesis.
- Discouraging differentiation of CD4+ T Cells into Regulatory T Cells by reducing the corresponding effect.

The use of such drugs varies in degree: how intensely they are applied determines the proportion of cells and interactions involved at each step. For example, when *ICI*s are used at their maximum, tumor cells completely (100%) lose their ability to bind to PD-1. Conversely, *TKI*s fully unmask the tumor, making it reveal itself to DCs. The DCs then phagocytize it and proceed with their antigen-presenting responsibilities. **Supplementary Materials** provide a systematic summary of these in **Supplementary Table 9**.

### 2.5 Real-Time and adaptive learning aspect

In the context of an agent-based learning model (ALM), the idea of learning plays a crucial role in improving the model’s ability to simulate real-world complexity. Learning, in this case, does not only refer to traditional machine learning algorithms, but more broadly to the model’s capacity to adjust and develop its behavior based on internal simulation dynamics or external, real-world information. To explain this, we propose a distinction between two related but conceptually separate learning aspects, Real-Time Learning and Adaptive Learning, each offering a different perspective through which an ALM can better represent a complex, real-world system:

*Real-Time Learning* refers to the model’s ability to perform dynamic and possibly localized adaptations during a single simulation run. It represents a form of internal learning that occurs within a simulation instance. This aspect mainly involves the interaction between an agent and its perceived environment: changes in the environment and the agent’s immediate surroundings are assessed to adapt current behavior according to a specific objective. Implementing a reinforcement learning algorithm to describe an agent’s behavior would fall into this category.

*Adaptive Learning*, differently from real-time learning which emphasizes adjustments during a single simulation, this method of learning pertains to inter-simulation or long-term improvements to the overall model. Instead of learning in one run, the model evolves over multiple simulations. This type of learning may involve a comprehensive update of simulation rules, parameters, or structural components based on external feedback, such as empirical data or analysis of simulation outcomes. From this, adaptive learning appears to be a stronger form of learning, similar to the traditional paradigm common in machine learning, as the model gradually aligns more closely with the unknown function observed. Ultimately, the goal of incorporating this aspect is to boost the overall accuracy and realism of the simulated function.

### 2.6 Tumor genetic adaptation

As a real-time learning example, we modeled tumor cell genetic evolution using principles from genetic algorithms. A Genetic Algorithm is a meta-heuristic inspired by natural selection, commonly used to generate solutions to optimization and search problems through biologically inspired operators such as selection, crossover, and mutation.

#### 2.6.1 Genetic representation

In our model, each tumor cell contains an internal string of nucleotides divided into multiple gene segments. These segments correspond to biologically important genes, and their roles are summarized in **Table 1**. Note that mutating a gene is not always beneficial for the tumor. The analyzed genes were selected based on well-established evidence demonstrating their mechanistic involvement as validated drivers in tumor immune evasion, genomic instability, cell cycle control, or oncogenic signaling.

**Table 1:** Overview of genes represented in the model grouped by genetic effects.

Behaviour	Responsible gene(s)	Notes	Ref.
*Antigen Presentation*	*MHC genes (e.g., HLA-A, HLA-B, HLA-C), JAK1, JAK2, B2M, TAP1, TAP2*	Mutation of MHC genes leads to poor antigen presentation and immune evasion. Loss-of-function mutations in JAK1, JAK2 lead to resistance to IFN-*γ* signaling and escape from immune killing. Loss of B2M renders MHC-I non-functional, preventing CD8+ T cell recognition. Loss of TAP1, TAP2 impairs peptide loading into MHC-I.	(24, 25)
*Checkpoint Pathways*	*CD274*	Overexpression of CD274, which encodes PD-L1, enables binding to PD-1 on T cells, suppressing their activity	(25, 26)
*Genomic Instability*	*BRCA1/2*	Inactivation of these tumor suppressor genes impairs DNA repair, leading to higher mutation rates and genomic instability	(27)
*Tumor Suppressor*	*TP53, RB1, CDKN2A*	These genes halt cell division or trigger apoptosis when active. Mutation or inactivation allows tumors to grow unchecked	(28–30)
*Proliferation*	*Oncogenes: MYC, RAS, EGFR, HER2*	When overactivated, these genes promote cell cycle progression and uncontrolled proliferation	(31–34)
*Division Reduction*	*APC*	When functional, APC suppresses cell division. Mutation leads to overgrowth due to loss of this control	(35)
*Peptide Mutation into* *Neoantigens*	-	Not driven by a single gene, but emerging from multiple mutations producing novel peptides (neoantigens) recognized by T cells	(36, 37)

**Figure 1:**
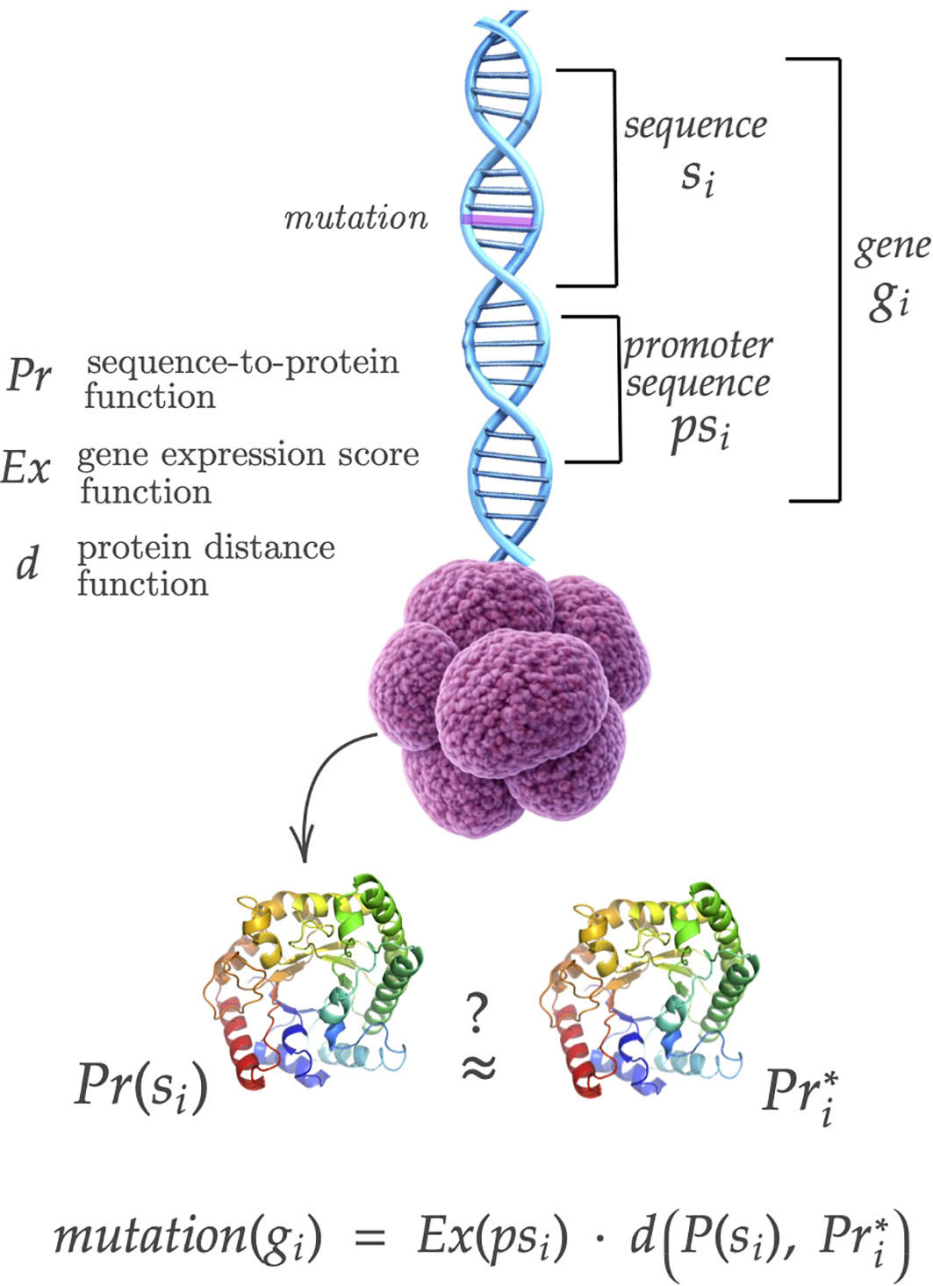
Model of the real-time learning component, conceived as the tumor genetic mutation process. Mutations in the gene sequence change the structure of the resulting protein. This representation allows us to encode both the behaviors of tumor cells (*gene sequence*) and their level of expression (*a promoter sequence*). DNA helix icon from https://it.pngtree.com/freepng/3d-blue-dna-helix-sequenceelement-isolated-on-transparent-background_18494507.html

As shown in **Figure 1**, each gene is represented by two parts:

*a promoter sequence* $p_{s_{i}}$ which encodes the regulation or expression level, influences how actively the gene is transcribed. A mutation in the promoter sequence affects this activity, thereby impacting how much of the corresponding protein is produced;

*a gene sequence* $s_{i}$, which is a string of DNA that encodes the structure and function of proteins.

Mutations in the gene sequence change the structure of the resulting protein.

This representation allows us to encode both the behaviors of tumor cells and their level of expression, consistent with biology. In this context, a mutated promoter sequence combined with a mutated gene sequence can enhance the overall mutated behavior, as an overexpressed gene produces more protein more frequently. To assess the mutation degree of the gene *g_i_*, we compare the deterministically produced protein $Pr(s_{i})$ with the corresponding *wildtype* protein, which represents the normal protein produced in the absence of any mutation. The expression level of the promoter sequence $Ex(p_{s_{i}})$ acts as a multiplier, amplifying the overall mutation effect.

#### 2.6.2 Fitness function

The fitness of a tumor cell is defined by its ability to survive. Mutations that offer a functional advantage in the current simulated environment (e.g., immune evasion, apoptosis resistance) increase the chances of that cell’s survival and reproduction.

For example, a mutation in the MHC gene may decrease antigen presentation on the cell surface, increasing immune evasion, and giving tumor cells more chances to proliferate. Similarly, a mutation in TP53 may block programmed cell death, allowing damaged or mutated cells to survive and divide. These mechanisms let tumor cells adapt to their internal states and interactions in real-time, based on local survival feedback.

### 2.7 Data-Driven simulation tuning

**Figure 2:**
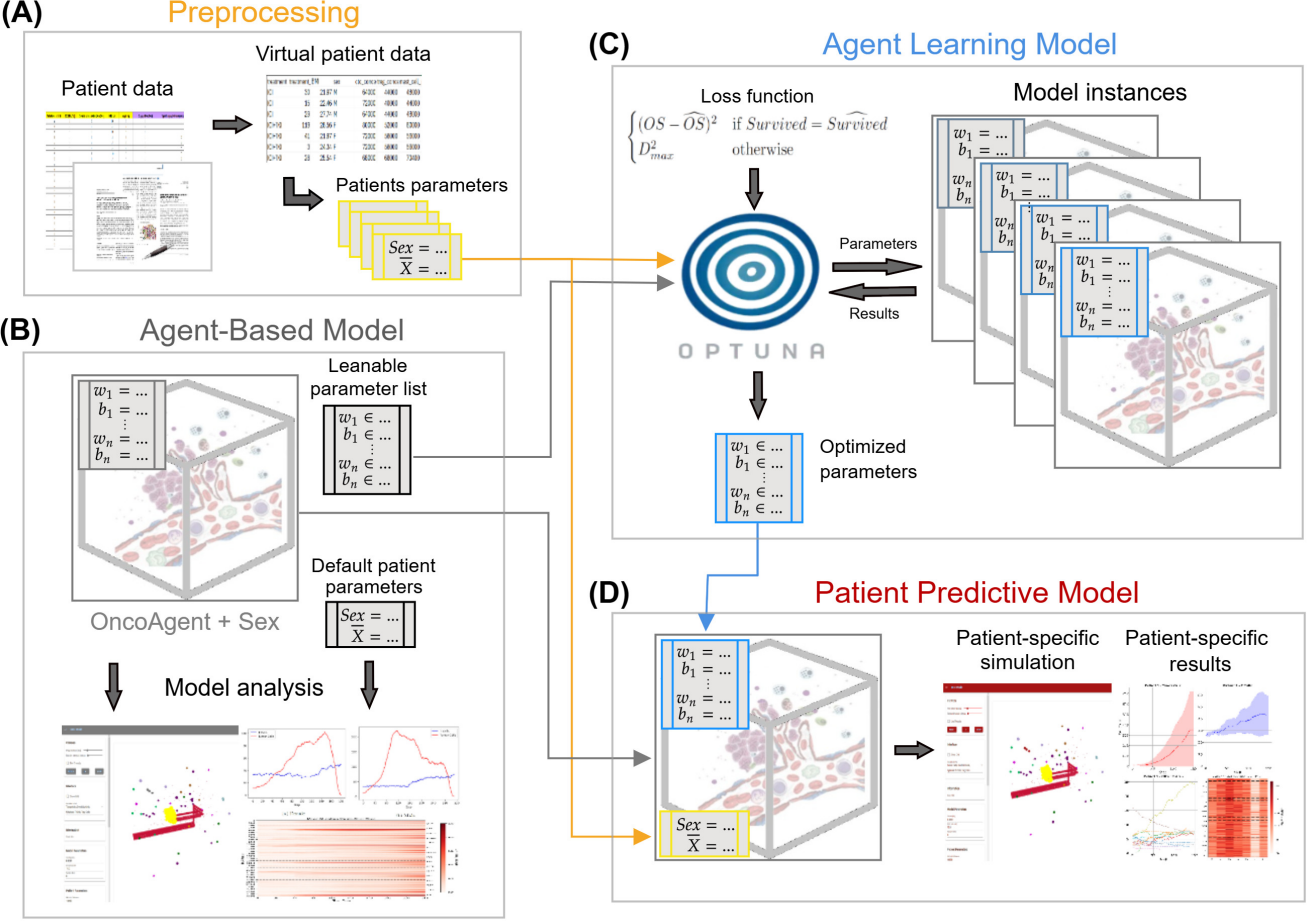
The architecture of the agent-based learning model designed for investigating the influence of sex hormones on therapeutic response in RCC patients. **(A)** The real-world clinical dataset pre-processed to extract individual patient outcomes and initial parameters derived from available clinical measurements and domain expertise. **(B)** The agent-based model (ABM), named OncoAgent in our previous work and implemented according to domain-knowledge of the biological system under study (14). Unobservable or unknown features of the real-world environment are represented as learnable model parameters. Initial patient-specific parameters are set based on literature and expert knowledge, and the baseline behavior of the simulated system is examined to refine the ABM design. **(C)** The proposed custom loss function used to guide the automated tuning of the learnable ABM parameters within a hyperparameter optimization framework OPTUNA. The model repeatedly instantiated and simulated for each candidate configuration and for each patient, potentially in parallel and with multiple random seeds. Simulation outputs are compared with real-world patient outcomes to iteratively update the learnable parameters and minimize prediction error. **(D)** The patient predictive model (PPM), an optimized model employed to predict therapeutic outcomes, either for patients included in the training dataset or for new, unseen patients. The model tuning procedure occurs jointly with the tumor’s genetic evolution within each individual simulation run, with each process dynamically influencing the other.

To enable adaptive learning, we equipped the model with a large set of tunable parameters. We designed our simulation process to resemble a supervised machine learning (ML) pipeline, where real-world clinical data guide the refinement of simulation parameters across multiple runs. This approach is illustrated in **Figure 2**, and **Supplementary Figure 2** in **Supplementary Materials**, and explained further in the following.

We use a curated set of clinical cases reporting structured patient data, such as biometrics, genetic markers, and observed clinical outcomes, including OS. Within the simulation, we assign learnable weights and biases to key dynamic variables—such as effects, perceived levels of sex hormones, interaction success, or the rate at which stochastic events occur in the environment, like angiogenesis. These weights indicate the degree of influence controlling how strongly a biological or environmental factor impacts simulation outcomes. For example, by normalizing tumor growth $e_{tg}$ and the additional tumor proliferation mutation $g_{MYC}$ within the interval *[0,1]*, we assign learnable weights and biases to compute the final probability of duplication in a single simulation step, *p_dup_* as:


$$p_{dup}=\{\begin{array}{c} 1\;\;\;\;\;\;\;\;\;\;\;if\;p_{dup}^{'}>1\\ 0\;\;\;\;\;\;\;\;\;\;\;if\;p_{dup}^{'}<0\\ p_{dup}^{'}\;\;\;\;\;otherwise \end{array}$$

$$p_{dup}^{'}=\frac{e_{tg}\cdot w_{1}+mutation(g_{MYC})\cdot w_{2}}{2}+b_{1}$$

Where $w_{1},w_{2}\in {\mathbb{R}}_{\geq 0},\;b_{1}\in \mathbb{R}.$

To assess the potential of adaptive learning in refining model behavior through learnable parameters, we incorporated real-world clinical data from the ARON dataset (2, 38). We selected a subset of RCC cases and conducted a manual preprocessing phase in which patient characteristics and tumor features were mapped to estimated concentrations of specific cell populations (more details are provided by **Supplementary Tables 1**, **S2** in **Supplementary Materials**). This step was necessary to ensure compatibility between the dataset features and our model inputs, while maintaining biologically consistent initial states for the simulations. For the adaptive optimization process, we used Optuna (39), an optimization framework widely used for hyperparameter tuning in machine learning. While versatile, Optuna is particularly well-suited for tasks involving large and complex search spaces, as in our case of parameter configurations. In our training setup, each trial run by Optuna represented a complete evaluation of the dataset, testing a unique combination of learnable parameters by simulating each record. At the end of each run, the *fitness function* is computed using the simulation results. In our case, we considered two possible functions based on the interpretation of the data:

*CI*: *concordance index* of the pair-wise relative order of all *OS* predictions among patients with respect to the sorting of real survival outcomes, defined as:


$$CI=\frac{1}{\left|P\right|}\;\sum_{(p_{i},p_{j})\in P}\text{Ι}(\hat{OS_{i}}<\hat{OS_{j}})$$

where *P* is the set of comparable pairs of patients, so that $(p_{i},p_{j})\in P\Leftrightarrow OS_{i}<OS_{j}\wedge \neg Survived_{i}$, while Ι(true)=1 and Ι(false)=0.

This metric is sensible in the uncensored interpretation of data: no exact outcome is observed for those patients $p_{i}$such that $Survived_{i}$ is true in observed clinical data, and all we know is that $p_{i}$ was still alive at time $OS_{i}$, preventing any comparison with patients having a greater *OS*.

*MSE*: *mean squared error* between the simulated and actual survival outcomes, defined as:


$$MSE(OS,Survived,\hat{OS},\hat{Survived})\;=\{\begin{array}{c} {\left(OS-\hat{OS}\right)}^{2}\;\;\;\;\;if\;Survived=\hat{Survived}\\ D_{\text{max}}^{2}\;\;\;\;\;\;\;\;\;\;\;otherwise\;\;\;\;\;\;\;\;\;\;\;\;\;\;\;\;\;\;\;\;\;\;\;\;\; \end{array}$$

where $D_{\text{max}}$ is the maximum possible difference between OS and $\hat{OS}.$ This metric is suitable and more precise when we have additional knowledge about survived patients and data incorporates definitive outcomes, such as confirmed recovery or successful termination of therapy.

After simulating with a specific parameter setup, we calculate the survival outcomes and compare them to real-world clinical data. The adaptive process aims to reduce the difference between simulated and actual outcomes. This is achieved by either minimizing the *MSE* or maximizing *CI* across the training dataset. By iteratively adjusting the learnable weights and biases, the system fine-tunes itself to improve predictive accuracy and biological plausibility. This enables the ALM to evolve over multiple runs, incorporating empirical knowledge and clinical evidence to enhance realism and generalization.

### 2.8 Ethical considerations

The ARON-1 project was approved from the ethics committee of the Marche Region (2021-492) and was performed in accordance with the Declaration of Helsinki. Due to the retrospective nature of the study, informed consent was not required.

## 3 Results

### 3.1 Sex-based differences in treatment response

**Figure 3:**
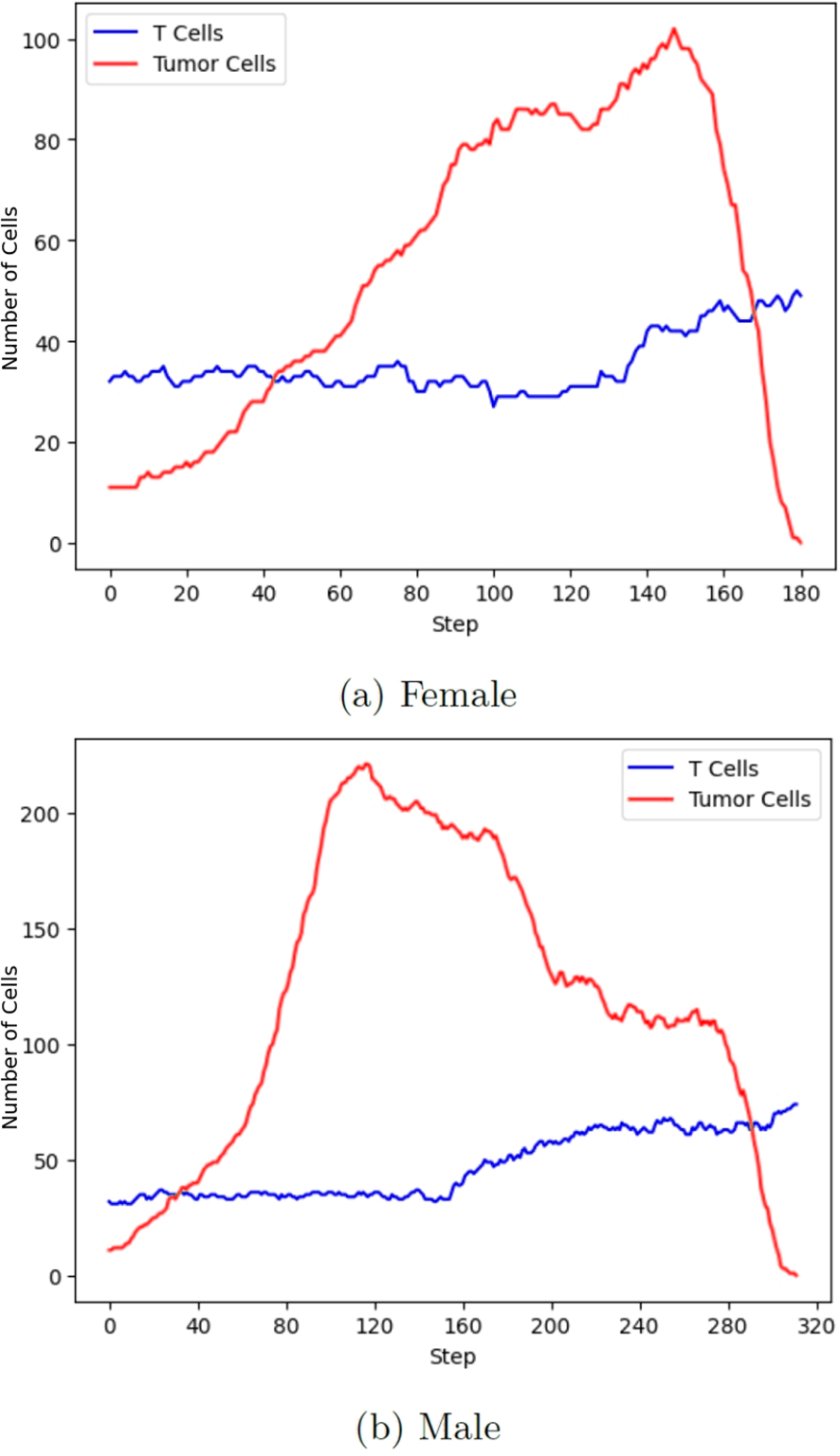
Cell counts from simulation runs using untrained models initialized with default patient parameters, with the *ICI+TKI* treatment start parameter set to 100. **(a)** Females tended to exhibit a weaker immediate response to the *ICI*-*TKI* combination therapy. This early resistance reflects patterns observed in real-world cases and could be due to lower initial engagement of immune mechanisms following ICIs administration. **(b)** Male subjects in the simulations displayed a more stable and consistently responsive system throughout treatment. Tumor progression appeared less volatile, and treatment efficacy, especially with ICI monotherapy, followed a comparatively steady course.

The simulation suggests clear sex-based differences in the progression of RCC and the effectiveness of treatment strategies, which appear broadly consistent with clinical observations (2), and as exemplified by the results shown in **Figure 3**. Within the model, females tended to exhibit a weaker immediate response to the *ICI*-*TKI* combination therapy. This early resistance reflects patterns observed in real-world cases and could be due to lower initial engagement of immune mechanisms following *ICI*s administration. However, the overall simulated innate immune response in females proved to be notably stronger over the longer term: increases in T Cells and other immune cells were often associated with marked declines in tumor cell numbers. When immune stimulation was sustained—mainly with support from *TKI*s— the model indicated more rapid tumor regression, significantly less immune cell exhaustion, and a higher likelihood of immune activation restoration in female patients. These findings suggest that, within the modeled framework, female immune system, while less reactive initially, may have the capacity to mount a stronger response under sustained stimulation. Conversely, male subjects in the simulations displayed a more stable and consistently responsive system throughout treatment. Tumor progression appeared less volatile, and treatment efficacy—especially with *ICI* monotherapy—followed a comparatively steady course. However, this apparent stability was paired with greater resilience of tumor cells, potentially reflecting more frequent or advantageous genetic adaptations over time. In this context, the *ICI* component appeared particularly important for male patients, as its absence often led to a diminished treatment effectiveness. Therefore, in males, long-term tumor control seems more reliant on the immune system’s ability to bypass tumor adaptations, highlighting the importance of therapies that target immune evasion mechanisms.

**Figure 4:**
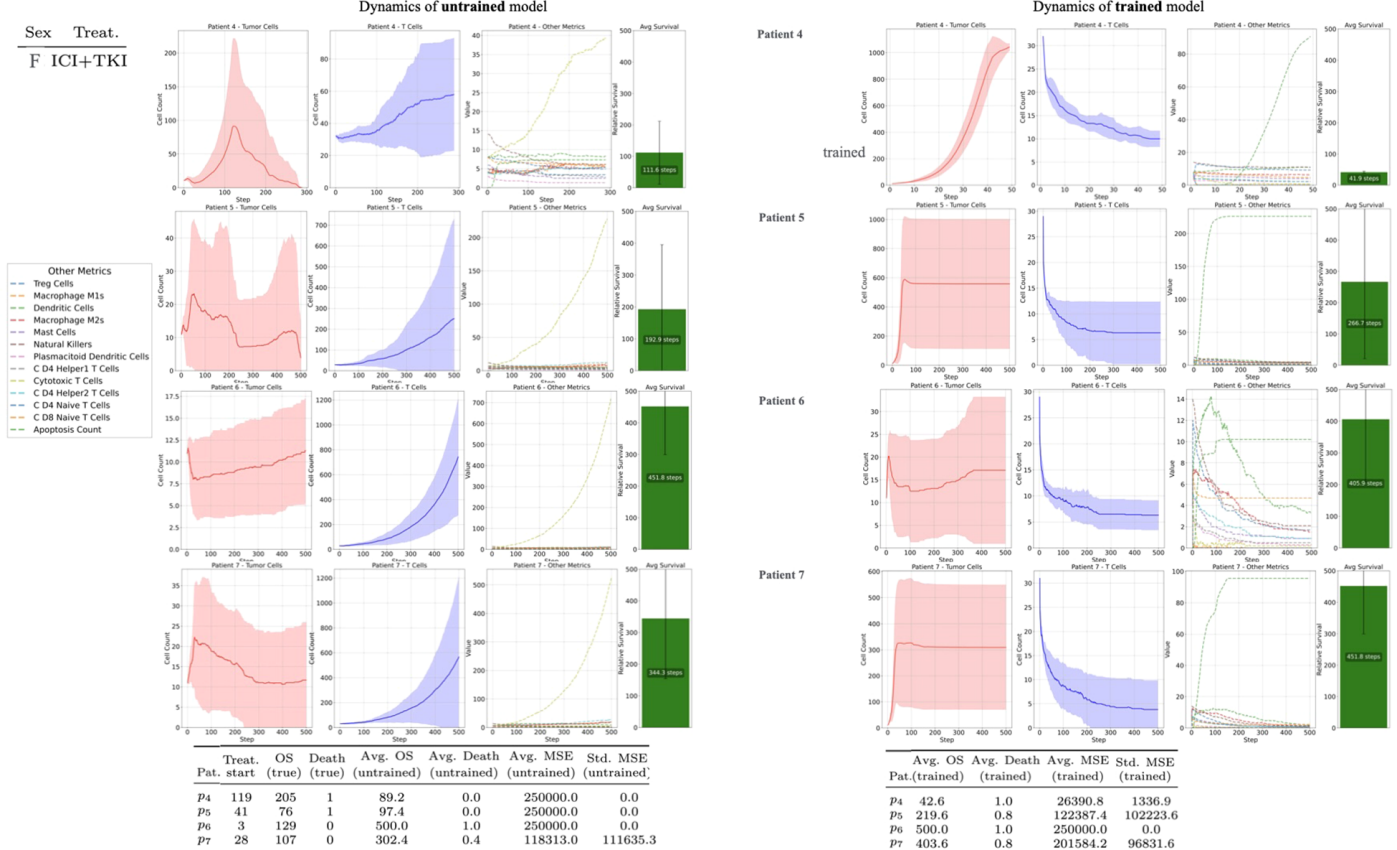
Female models derived from the analysis of four female patients, the two line-graphs correspond to female virtual images and depicting mean cell counts across 10 simulation seeds for the untrained and *MSE*-trained models. Solid curves represent the mean trajectory, while dashed lines indicate the associated standard deviations. For each patient $p_{4},p_{5},p_{6},p_{7}$, the plot also displays the average overall survival (OS) time insimulation steps.

In line with the nature of agent-based systems, we interpret these observed sex-specific differences as reflecting the intrinsic biological structure encoded in the model, as dynamics emerge from nonlinear feedback loops and cumulative interaction history between agents – for instance, tumor genetic adaptation, immune exhaustion and therapeutic modulation. For these reasons, stochastic components and model initialization contribute variably without solely determining the qualitative trends. For instance, as can be seen later on from results in **Figure 4**, a delayed yet stronger immune response has been observed as a consistent trend in differently initialized female instances of the model and across differently seeded simulations of the untrained model. Nevertheless, these specific results should be interpreted as exploratory and hypothesis-generating. They illustrate how the model can reproduce clinically plausible sex-specific patterns and may provide a basis for further refinement and validation against empirical data.

### 3.2 Genetic real-time adaptation of tumors

As a result, regarding the real-time learning aspect, the simulation enables tracking of how tumor mutations gradually accumulate over time in response to sustained immune pressure. An example is shown in **Supplementary Figure 9** of **Supplementary Materials**, illustrating the average mutation mask calculated across all tumor cells during a sample simulation in which tumors were continuously targeted by Cytotoxic T cells.

In this context, the mutation mask refers to a binary bitmask that marks each nucleotide as either mutated (1) or non-mutated (0). By averaging this mask across the entire tumor population, we obtain a profile that reflects the percentage of cells carrying a mutation at each nucleotide position. This metric highlights the evolutionary pressure on specific genomic regions and their resulting variability across the tumor population. The sample data shows how some genes are more prone to mutations than others. For example, Breast Cancer-Associated Gene 1 (BRCA1), a gene known for its role in DNA repair, exhibits frequent mutations. This likely contributes to an increase in transcriptional errors and overall genomic instability. Similarly, CD274, which encodes the PD-L1 protein involved in immune evasion via the PD-1/PD-L1 checkpoint pathway, also shows a high mutation rate—possibly reflecting selective pressure to improve immune escape. Note that, in contrast, other genes such as Epidermal Growth Factor Receptor (EGFR) demonstrate a lower mutation frequency. This may reflect the fact that mutations in EGFR are disadvantageous under the simulation conditions. Indeed, when mutated, EGFR may impair proliferative signaling pathways, reducing tumor fitness, and therefore being expelled in the evolutionary process.

### 3.3 Patient-adapted simulation

The real-world tumor clinical data from the ARON dataset, available on Zenodo^1^ platform, consists of 123 patient records. From this collection of data, we manually filtered the subset of patients affected by RCC as part of the preprocessing, consisting of 7 subjects—3 males and 4 females. For each patient, we construct their virtual image, for both personal characteristics and cancer features, which provides the estimation of specific cell concentrations.

For preliminary results on adaptive learning, we used the seven virtual images to update the learnable parameters and refine the model’s behaviour.

We conducted a total of 26 optimization trials with each fitness function, enabling the system to iteratively converge toward more accurate configurations. The **Supplementary Materials** show more details in **Supplementary Figure 3**. The adaptive learning procedure yielded promising improvements in both training procedures: the optimized parameter sets achieved a significant reduction in prediction *MSE* compared to the baseline results obtained using manually tuned defaults, while the trend of CI over trials shows increasing stability towards fitter configurations of parameters. This testifies the feasibility and potential of data-driven methods to align simulation outputs with clinical observations, thereby enhancing the predictive power and realism of the ALM.

**Figure 5:**
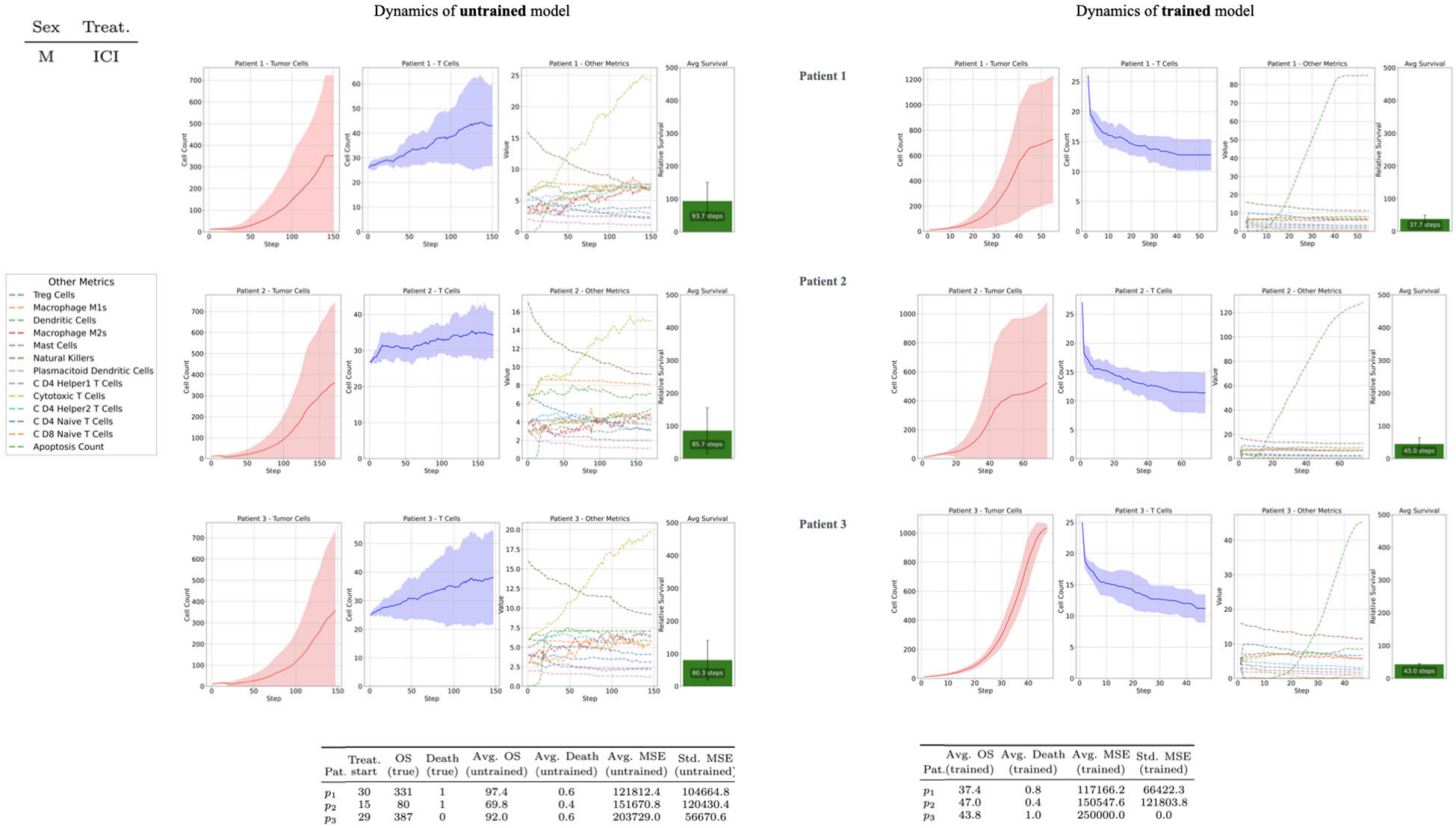
Male models derived from the analysis of three male patients, the two line-graphs correspond to male virtual images and depicting mean cell counts across 10 simulation seeds for the untrained and *MSE*-trained models. Solid curves represent the mean trajectory, while dashed lines indicate the associated standard deviations. For each patient $p_{1},p_{2},p_{3},$ the plot also displays the average overall survival (OS) time insimulation steps.

**Figure 6:**
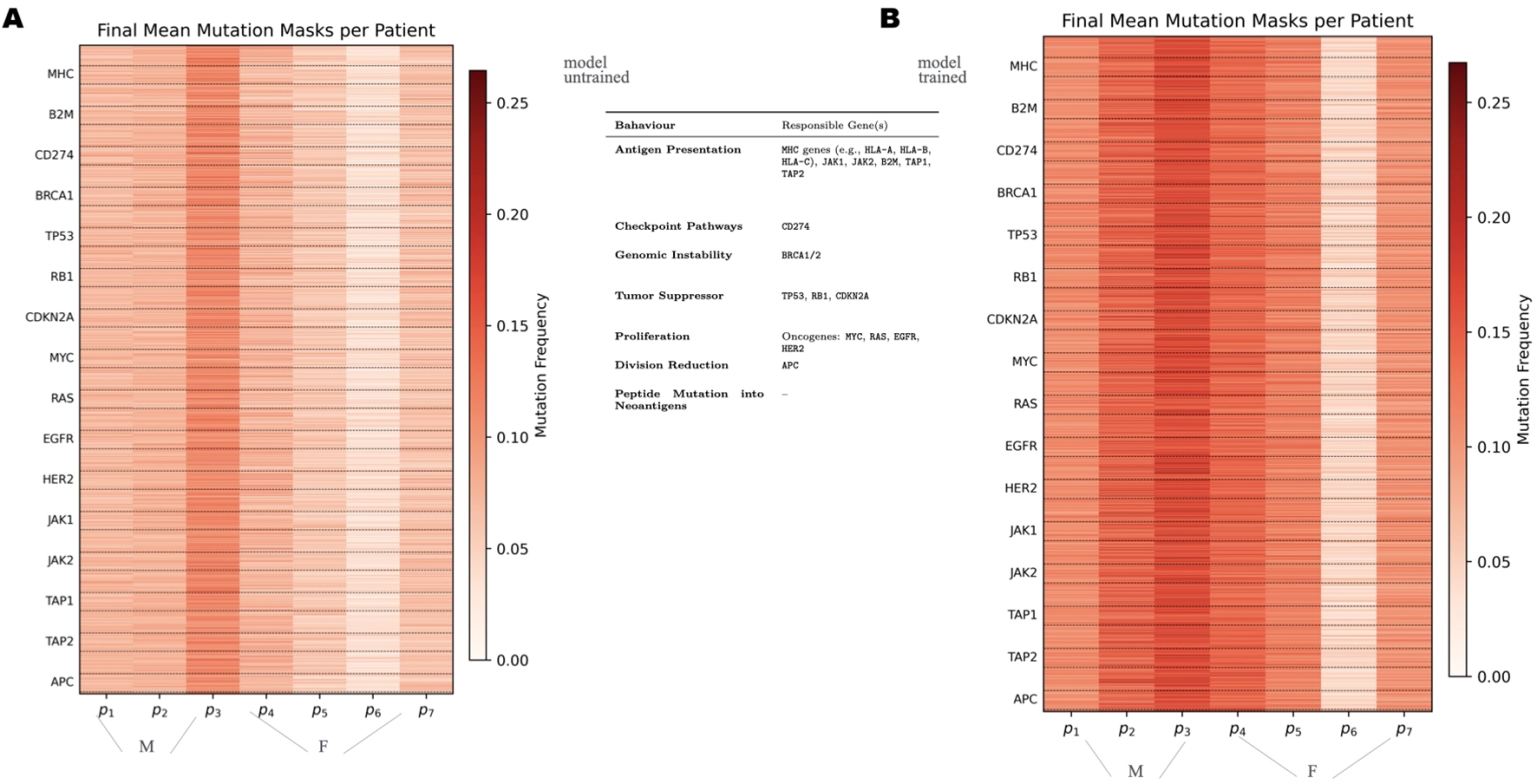
Panels **(A, B)** illustrate the final average mutation mask for the seven patients in the untrained and trained settings, respectively, indicating the distribution of gene mutations across seeds at the end of the simulations shared in the legend.

**Figures 4**–**6** show the simulated function *f* for each patient selected from the ARON dataset. However, as shown in **Table 2**, data from individual patients — gathered across additional simulation runs of both *MSE-*trained and untrained versions of the model — indicate that even a small number of training iterations can produce parameter configurations that reduce differences between observed and trained outcomes. For instance, patients such as $p_{4}$and $p_{5}$show better agreement between simulated and observed survival outcomes. This effect may be due to changes in high-impact parameters, such as those influencing the base tumor growth rate. For these same patients, however, we observe increased standard deviation on the *MSE*. While suggesting an unstable model configuration in terms of predicted OS, we believe this is partially due to the optimization dynamic privileging aligned average values to the expense of stability. In this context, while stochastic amplification induced by changes in high-impact parameters is worth considering, the role of biological heterogeneity among patients is a plausible alternative explanation, since patients that remain well-classified (e.g. $p_{1}$) show a reduction in their standard *MSE* deviation instead. These results highlight that the selection of learnable parameters and their allowed flexibility during training is nevertheless critical and should be guided by domain experts. To this extent, we believe sensitivity analysis of high-impact parameters could serve as a powerful tool for determining the causal impact on simulation outcomes and their appropriate ranges shaping the optimization search space. Refer to **Supplementary Table 9** in **Supplementary Materials** for more preliminary insights into the statistical associations between manipulated parameters and the evaluated error metrics. With the exception of the importance of the Natural Killers’ kill rate parameters, which shows associations with the observed errors, no parameter manipulation appears to be consistently associated with both error metrics. For this reason, we leave the detailed investigation on single-parameter sensitivity analysis as a worthy effort for future works involving a larger set of available data.

**Table 2:** Numerical results on simulation outcomes from the untrained and trained models of the seven patients.

Patient	Sex	Treat.	Treat. start	OS (true)	Death (true)	Avg. OS (untrained)	Avg. Death (untrained)	Avg. MSE (untrained)	Std. MSE (untrained)	Avg. OS (trained)	Avg. Death (trained)	Avg. MSE (trained)	Std. MSE (trained)
p1	M	ICI	30	331	1	97.4	0.6	121812.4	104664.8	37.4	0.8	117166.2	66422.3
p2	M	ICI	15	80	1	69.8	0.4	151670.8	120430.4	47.0	0.4	150547.6	121803.8
p3	M	ICI	29	387	0	92.0	0.6	203729.0	56670.6	43.8	1.0	250000.0	0.0
p4	F	ICI+TKI	119	205	1	89.2	0.0	250000.0	0.0	42.6	1.0	26390.8	1336.9
p5	F	ICI+TKI	41	76	1	97.4	0.0	250000.0	0.0	219.6	0.8	122387.4	102223.6
p6	F	ICI+TKI	3	129	0	500.0	1.0	250000.0	0.0	500.0	1.0	250000.0	0.0
p7	F	ICI+TKI	28	107	0	302.4	0.4	118313.0	111635.3	403.6	0.8	201584.2	96831.6

The first columns contain relevant specifications for simulation setup derived from the real-world data, indicating the sex (Sex) of each patient, the applied treatment (Treat.) and the simulation step at which the treatment is introduced (Treat. start). Following, columns marked (true) indicate the ground-truth simulation outcomes, specifying the *OS* and whether the patient survived (*0*) or died (*1*). These are also derived from the ARON dataset. The remaining columns represent statistics about simulation outcomes across seeds in both models (untrained, trained), specifically the average of binary death outcomes (Mean Death), the average mean square error (*Avg. MSE*) and its standard deviation (*Std. MSE*) across seeds.

### 3.4 Code availability

Source code for the MAS simulation and learning model is available in the repository link: https://github.com/marco-caputo/RCC-agent-learning-model

Source code has been also placed on the Zenodo platform at the following address: https://doi.org/10.5281/zenodo.18727138

The pseudo-code of the genetic algorithm simulating promoter and gene mutations, with fitness defined by immune evasion and proliferation advantages during the tumor evolution, is described by **Algorithm S1 in Supplementary Materials**.

## 4 Discussion and conclusions

Sex-based differences are increasingly recognized as key factors influencing how patients with RCC respond to immune therapies. Variations in tumor biology, antitumor immunity, and pharmacokinetics between males and females may affect tumor immunogenicity, the makeup of the tumor microenvironment, and treatment outcomes, including side effects (1, 2, 40, 41). Large meta-analyses and broad reviews of *ICI*s suggest that males often gain slightly more benefit from *ICI*s than females, although this effect is small and varies across different tumor types and treatment settings (41, 42). Conversely, women tend to experience higher rates of immune-related adverse events, supporting the idea that sex is a biologically significant, yet complex, factor influencing immunotherapy effectiveness and tolerability (1, 43, 44).

From an immunobiological perspective, females generally mount stronger innate and adaptive immune responses, exhibit higher circulating levels of several cytokines, and have distinctive patterns of tumor mutational burden and antigen presentation compared to males (6, 8, 41, 42). These differences likely influence sex-specific patterns of *ICI* sensitivity and immune-related toxicity. In RCC specifically, epidemiological data confirm a higher incidence in men and more aggressive disease at presentation, while immune profiling suggests that female patients may have greater baseline immune competence but also a higher tendency toward immune dysregulation and autoimmunity (2, 10, 41). Recent real-world and prospective studies add complexity: in a multicenter cohort of advanced RCC treated with first-line *ICI* combinations, or also tyrosine-kinase inhibitors (*TKI*) alone, female sex was linked to shorter progression-free survival in some *ICI*-based regimens but not associated with worse overall survival, indicating that subsequent therapies and long-term immune reactivation could offset sex-related differences in early disease control (2, 45). These varied findings underscore the need for mechanistic models that transcend simple male–female distinctions and explicitly incorporate hormone signaling, immune cell states, and tumor evolution over time.

In this context, our RCC-specific ALM integrates sex hormone modulation, tumor genetics, and PD-1/PD-L1 dynamics into a spatially explicit model of the tumor microenvironment. Simulations from our model suggest that males may show more stable and sustained responses to immune-based combinations than females, despite females having inherently stronger immune activation potential. This pattern aligns with recent single-cell and translational studies indicating that male RCC is marked by higher infiltration of CD8+ T cells, which are nonetheless trapped in a deeply exhausted, androgen-driven state (46–48). In a large single-cell transcriptomic analysis, androgen–androgen receptor (AR) signaling promoted CD8+ T-cell dysfunction and was linked to poorer outcomes in male RCC patients receiving *ICI*s, while preclinical data suggest that AR blockade can partially restore CD8+ T-cell function and synergize with PD-1/PD-L1 blockade (46). Additional work in mouse models and human cohorts has demonstrated that AR signaling inhibits cytotoxic gene programs and promotes exhaustion-like states in CD8+ T cells, contributing to sex-biased antitumor immunity across various tumor types (47, 48). These findings support one of the central assumptions of our model: that androgen-dependent pathways in males can weaken effective immune surveillance unless specifically targeted, thus influencing both early treatment responses and long-term tumor control.

Beyond T cells, multiple lines of evidence now emphasize sex hormone pathways as key prognostic and predictive factors in RCC. Recent reviews and translational studies describe how estrogen and androgen receptors interact with Hypoxia-inducible factor (HIF), Vascular endothelial growth factor (VEGF), and metabolic signaling, affecting RCC initiation, angiogenesis, and immune escape (10, 11, 49). A newly proposed “sex-hormone–related gene” (SHAG) signature combines hormone-related gene expression with immune and metabolic pathways, grouping clear-cell RCC patients into risk categories with significantly different survival outcomes (50). Additionally, emerging data link circulating sex hormone levels and sex-specific survival mutations to RCC prognosis and treatment response, highlighting that the hormonal context is not just a background variable but an active, evolving driver of disease progression (2, 49, 50). Our ALM explicitly models hormone-dependent regulation of CD8+ T-cell activation, apoptosis, and killing efficiency, as well as antigen presentation thresholds. This mechanistic approach provides a platform to investigate how perturbing androgen or estrogen signaling—alone or in combination with PD-1/PD-L1 and VEGF-targeted therapies—may alter tumor–immune interactions in a sex-specific manner.

Systemic metabolic state and body composition, reflected in our model through BMI and adipocyte-related signaling, represent additional layers of sexual dimorphism that may influence immunotherapy outcomes. Sex differences in cachexia, adipokine signaling, and immunometabolism have been observed across various cancer types, with female and male patients displaying distinct patterns of muscle loss, inflammatory cytokines, and hormone–metabolic crosstalk during treatment (14, 42). These systemic factors interact with tumor-intrinsic and immune parameters, supporting our choice to include BMI as a key input in the simulated function f(Sex, X). By incorporating sex hormones, immune cell abundances, and metabolic proxies, the ALM facilitates hypothesis generation about how combinations of *ICI*, *TKI*, endocrine modulators, and lifestyle interventions might benefit male and female RCC patients differently - hypotheses that are difficult to test solely through conventional statistics on retrospective cohorts.

Our work also fits within a rapidly evolving field of mathematical and agent-based modeling of tumor-immune interactions. Agent-based models have become powerful tools for capturing spatial heterogeneity, clonal competition, and immune escape strategies, complementing ordinary differential equation models and systems of partial differential equations (10, 13, 15, 50, 51). Recent ABM applications have explored how microenvironmental structure, cell-to-cell communication, and extracellular matrix remodeling influence the success of immune checkpoint blockade and cytotoxic chemotherapy in solid tumors (41, 51, 52). Simultaneously, new frameworks for calibrating and validating ABMs emphasize the importance of rigorous parameter estimation, identifiability analysis, and using high-dimensional single-cell or imaging data for model fitting (51, 53). Our adaptive learning pipeline, which combines an ABM with supervised machine learning and hyperparameter optimization using Optuna, aligns with these trends by treating clinical data not just as static inputs but as drivers of iterative model refinement and parameter learning.

Nevertheless, the limitations of our study reflect broader challenges in the field. First, the scarcity of large, high-quality, sex-annotated RCC datasets with detailed immunophenotyping and hormonal measurements limits both model development and validation. While real-world registries provide robust clinical outcomes and baseline characteristics, they rarely include longitudinal immune cell profiling, cytokine panels, or serial hormone levels, which are increasingly seen as sex-specific biomarkers of response to *ICI*s (2, 38, 42, 45, 53). Second, running many stochastic simulations for each parameter configuration involves substantial computational costs, especially when modeling multi-scale processes (genetic evolution, cell-to-cell interactions, drug pharmacodynamics). As recent ABM methodology reviews highlight, addressing parameter identifiability and uncertainty quantification requires thoughtful surrogate modeling, dimensionality reduction, and parallel computing strategies (44, 45, 54) so as a more flexible adaptability paradigm suitable to capture the evolution of the model entangling the agent interaction matrix to the microenvironment (55). Finally, our current adaptive learning procedure optimizes parameters based on endpoints such as OS and binary survival status. Expanding the loss function to include intermediate tumor and immune trajectories (e.g., serial imaging, circulating lymphocyte subsets, cytokine patterns) could improve biological accuracy but requires more detailed clinical data than are currently accessible.

Although these limitations exist, our ALM provides a conceptual and computational framework for studying how sex hormones, immune responses, and tumor evolution interact to influence responses to immunotherapy in RCC. By integrating real-world data from international registries with mechanistic models of hormone–immune–tumor interactions, the model generates testable hypotheses about why males and females respond differently, how androgen- or estrogen-targeting therapies might work with *ICI*s and *TKI*s, and which patient groups could benefit most from sex-specific treatment adjustments. Future research should focus on refining the model with prospective data that include sex-specific immune and hormonal profiles, exploring combined therapies that directly target androgen-CD8+ T-cell exhaustion pathways in male RCC, and using the ALM as a virtual trial platform to guide the design of sex-specific clinical trials and biomarker research in advanced RCC. Ultimately, gaining a clearer mechanistic understanding of sex-based immunobiology could improve risk assessment, treatment options, and help reduce outcome disparities for both men and women with RCC.

## Data Availability

The datasets presented in this study can be found in online repositories. The names of the repository/repositories and accession number(s) can be found in the article/**Supplementary Material**.

## Footnotes

1
https://zenodo.org/records/13353313
