# Supplementary material for: An agent-based learning model integrating sex differences in renal cell carcinoma
Source: Front Immunol. 2026 Apr 1;17:1779638. doi: 10.3389/fimmu.2026.1779638 (PMC13126258; doi:10.3389/fimmu.2026.1779638)

**Supplementary Materials to**

***An Agent-Based Learning Model Integrating Sex Differences in Renal Cell Carcinoma***

In this document we describe in more detail the applied learning procedures along with the environmental biological components and their interactions which discriminate the biological function respect to patients’ sex. As this work extends the original *OncoAgent* simulation framework, we describe here the introduced entities and mechanisms, mainly involving immune–tumor interactions and sex-specific effects within the tumor microenvironment (TME). This includes the relevant modelling aspects introduced in our version of the model, related to modelling choices and algorithmic behavior which details are not addressed in the main dissertation. More implementation details regarding unchanged aspects inherited from *OncoAgent* can be found in Supplementary Material of our previous work [14].

1. ***Tumor Microenvironment and Learning Aspects***

As described in **Figure S1**, a tumor grows in a surrounding environment (tumor microenvironment) made by other cells, among which we can identify different types of immune cells as listed in **Table S1**.

As schematized in **Figure S2**, the function $f$ entailing such evolution and the corresponding immune response is parametrized with the set of parameters $\theta$ described in **Table S2**. The adaptive learning aspect is enabled by using mapped real-world inputs $\vec{x}$ for initializing the model (as described in the following paragraph) and comparing the output $\hat{y}$ with the ground truth $y$ over different configurations of parameters. Values of $\theta$ are iteratively refined according to observed simulated outcomes to reduce a measure of error of interest over training trials (or epochs) of simulations. Each trial corresponds to a candidate parameter configuration $\theta$. During a trial, one or more differently seeded simulations are executed under the same parameter setting for each patient. Different seeds may be used to account for intrinsic stochasticity of the model. The resulting outcomes are aggregated to compute the trial-specific error value. The sequence of trial error values obtained across 26 optimization trials is reported in **Figure S3** and compared against the baseline error obtained using the uncalibrated parameter configuration. Preliminary insights into the statistical associations between manipulated parameters and the evaluated error metrics during such procedures are shown in **Table S3**. All results are shown separately for each adopted error metric.

On the other hand, the real-time adaptive behavior of the tumor within each simulation run is formalized through the high-level pseudocode specification provided in **Algorithm S1**. Note that this adaptive process does not correspond to an external parameter optimization step; but rather emerges from stochastic interactions among agents and from the rule-based behavioral dynamics governing individual tumor cells and genetic influences. An exemplifying result of the real-time learning aspect is depicted in **Figure S4**, illustrating the evolution of the average mutation mask calculated across all tumor cells during a sample simulation in which tumors were continuously targeted by Cytotoxic T cells.

1. ***Mapping of the* ARON *dataset to virtual patients***

The ARON dataset is mapped to individualized virtual patients by translating clinical and immunogenomic features into initial conditions of the agent-based simulation. The complete list of such initialization parameters are listed in **Table S4**. Specifically, patient-level variables are converted into baseline immune concentrations that define the initial simulation state, in accordance with the default cells concentrations defined for the OncoAgent simulator. **Table S5** presents the scoring system linking available patient data to the cell concentrations used to initialize the simulated environment. Provided scores are heuristic and based on published immunogenomic and clinical studies in RCC to support our proof-of-concept. In principle, they could be derived more systematically from statistical analyses correlating these features with biopsy results. This mapping is applied in **Table S1**, where the positive and negative associations inform estimates of concentrations as deviations from the standard values. Finally, the mapped cell concentrations are integrated into the full data conversion procedure summarized in **Table S6**. This table details both the numerical values assigned to each column variable of the virtual patient image dataset and the corresponding rules governing their derivation.

1. ***Sex-dependent differences***

Based on evidence reported in the reference studies [2, 32], sex-dependent differences in immune and tumor composition are incorporated at simulation initialization. These are reflected as gradual adjustments to concentrations of selected agents and to the probabilistic generation of sex hormones within the tumor microenvironment. Specifically, a gradual redistribution of CD8⁺ T cells is applied during the first simulation steps through a bounded drift mechanism. This drift scales the CD8⁺ population up to ±25% over a fixed number of iterations, increasing counts in males and decreasing them in females. This procedure introduces a controlled early divergence between sexes while avoiding abrupt or deterministic shifts. On the other hand, sex-dependent hormone spawning probabilities used throughout the whole simulation are summarized in **Table S7**.

1. ***Influence of Sex-hormones***

Sex hormones (Estrogen, Progesterone, and Testosterone) are explicitly modelled as passive agents within the TME but don’t exhibit behavior outside movement. Hormones originate from blood vessels, at production rates dependent on the global Sex parameter, and diffuse into the microenvironment. They are not generated exclusively at initialization but are produced dynamically at each time step in randomly selected blood-occupied grid locations. Their movement follows a stochastic drift process biased away from blood vessels, simulating spatial dispersion within the TME. Their impact on the behavior of CD8+ T Cells is regulated by the learnable parameter $w_{sex\_hormone\_CD8}$. As shown in detail in **Table S8**, in this formulation estrogen promotes cytotoxic activity and proliferation while reducing apoptosis, whereas testosterone and progesterone exert inhibitory or pro-apoptotic effects. Perceived hormone signals are exponentially decayed by a factor of 0.9, and perceived hormones are instantly consumed upon perception. In this way, observable effects on delays of immune response and its effectiveness are not imposed by other interventional mechanisms (e.g. threshold-based hormone switching, hard-coded responses) but rather driven by hormonal influences on T Cells behavior through continuous modulation.

These hormonal influences, in our model, are also implemented in the case of CD4+ T cells, which are necessary for orchestrating the immune response. They play a role in the regulation of the immune response either by specializing as regulatory T cells or helper cells TH1 and TH2, and these specializations depend on learnable parameters. The degree to which they are affected is dependent on a weighted concentration of the surrounding sex hormones and they yield different influences at different concentration thresholds.

1. ***Modelling of Treatment***

Treatment has been diversified into the application of *ICI*, *TKI* or a combination of the two. Each single drug can be applied with a proportion ranging from 0 to 1, indicating intensity of administration. Treatments are not considered agents, as they are not situated into the TME. However, their influence is defined in a stepwise manner according to their effectiveness $f$, computed as the product between the proportion and the correspondent learnable weight parameter. **Table S9** indicates the list of such influences for each drug. In the current version of the model, applying any of the two drugs in isolation, produces the indicated effects with a proportion of 1, starting from the first time step when the treatment starts. The combination of both drugs consists in collecting both lists of effects each with a proportion of 0.5.

1. ***Agent’s algorithmic behavior***

The behavior of several pre-existing agents was revised to incorporate additional biological mechanisms. In particular, substantial modifications were introduced for Tumor Cells and all T Cell families. The agents whose stepwise algorithmic behavior differs from the original model are illustrated in **Figures S5–S9**. At each simulation step, all T Cells now dynamically adjust their internal functional parameters in response to locally perceived sex hormone concentrations, allowing hormone-dependent modulation of proliferation, apoptosis susceptibility, and effector activity. Cytotoxic T Cells additionally experience both natural and tumor-induced exhaustion and may undergo PD-1/PD-L1–mediated inhibition with a probability determined by tumor mutational status. Furthermore, CD4⁺ and CD8⁺ T Cells no longer respond to whole tumor cells directly but instead receive tumor-derived, mutation-dependent antigens. Activation occurs only if the presented antigen is not classified as self and if the Hamming distance between the antigen sequence and the T Cell receptor lies within a predefined recognition threshold.

1. ***Other aspects***

Our new model version has been re-designed to support longer simulations. To this extent, to prevent uncontrolled exponential growth of proliferating tumor and immune populations, a natural death mechanism was introduced for all cell types. At each simulation step, every cell has a small probability of spontaneous apoptosis governed by the dedicated weight parameter $w_{cell\_base\_death\_prob}$.

**DATA AVAILABILITY**

The data and codebase produced by our study, which enable reproducibility, are available at:

**Repository link:** https://zenodo.org/records/18727138

**DOI:** 10.5281/zenodo.18727137

The original clinical dataset is available at:

**Repository link:** https://zenodo.org/records/13353313

**DOI:** 10.5281/zenodo.13353312

**FIGURE LEGEND**

**Figure S1.** A schematic version of the *simulated tumor microenvironment* that shows in the user interface blood vessels that carry oxygen and hormones, and depicts angiogenesis, which promotes tumor spread.


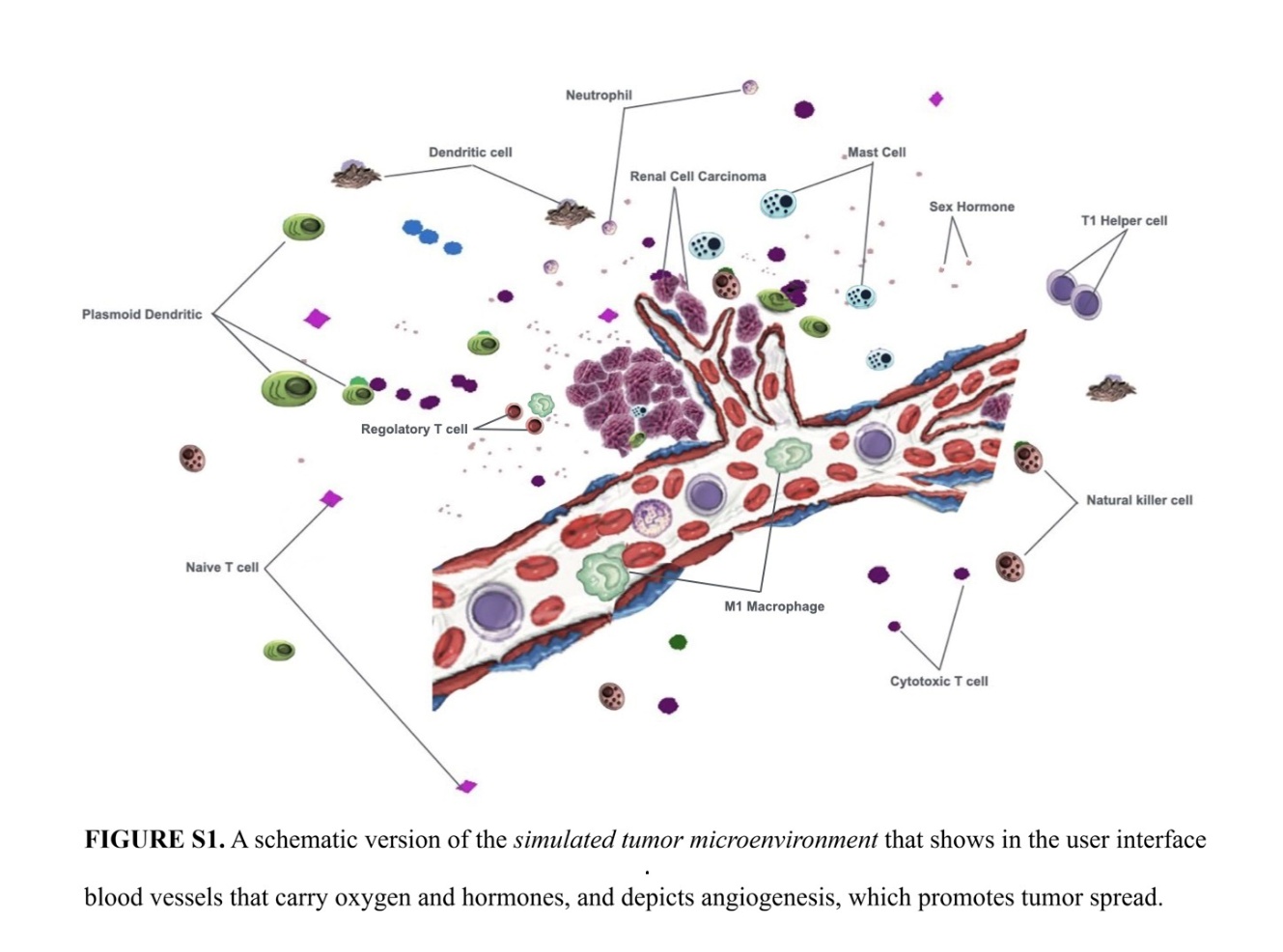


**Figure S2.** Graphical description of the data-driven adaptive learning approach.


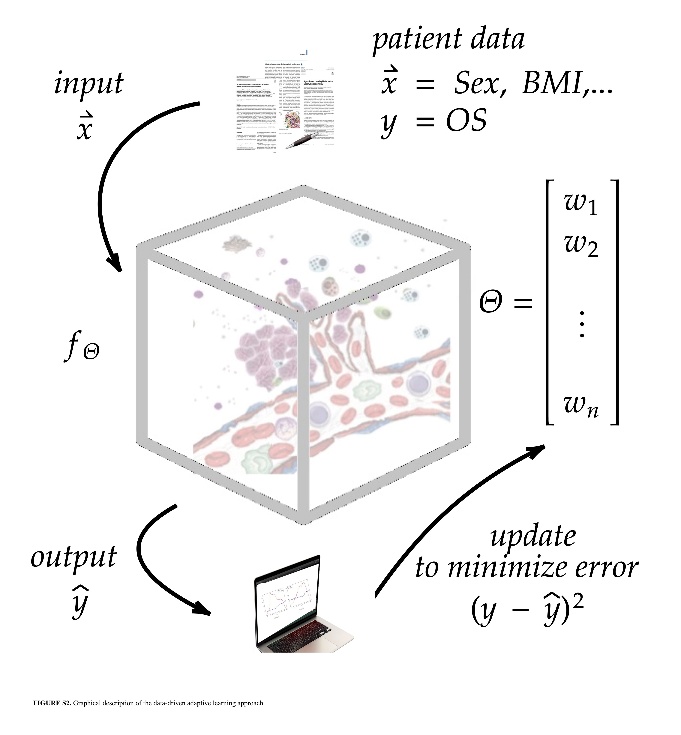


**Figure S3.** Trends of the intermediate errors during the two adaptive learning training processes. **
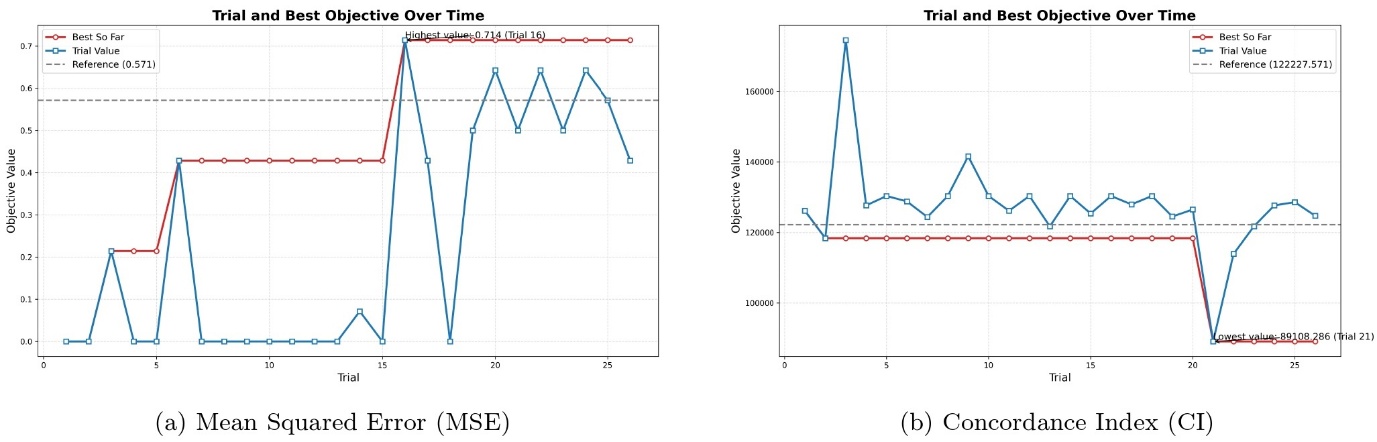
**

**Figure S4.** Evolution of the average mutation mask of tumor cells in a sample selection-intensive simulation.


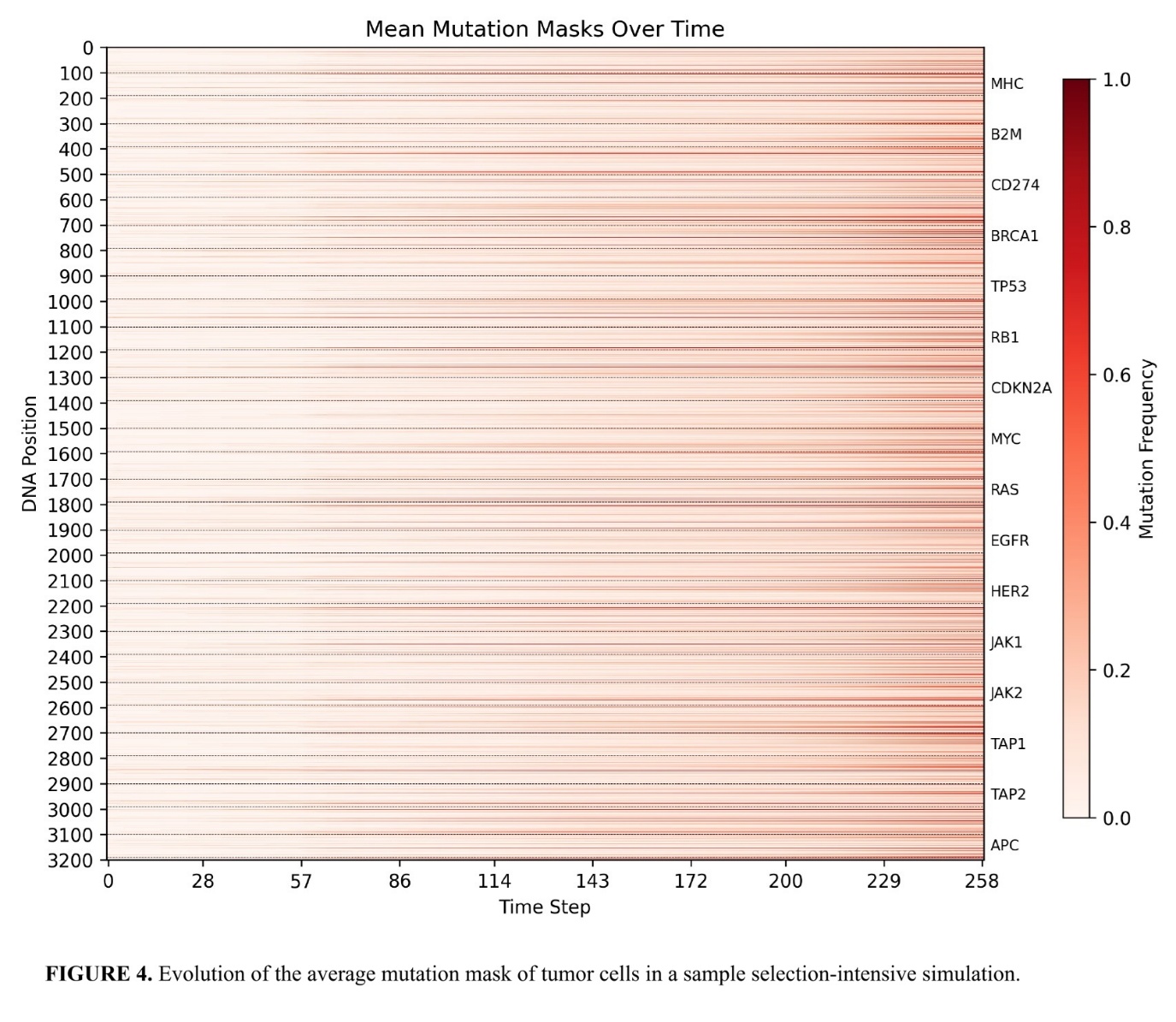


**FIGURE S5.** Behavior of a Tumor Cell at each time step of the simulation. At every step, the Tumor Cell first evaluates apoptosis, which depends on the experienced Tumor Apoptosis Effect, baseline apoptosis bias, and DNA-driven suppression mechanisms. If apoptosis is triggered, the cell is removed from the model and, in the presence of TKI treatment, may release neoantigens that can be processed by nearby Dendritic Cells. If the cell survives, it evaluates duplication. The probability of duplication depends on the experienced Tumor Growth Effect, DNA-driven proliferation potential, baseline growth bias, and, if blood access is available ($B$=1), an additional contribution mediated by Angiogenesis Effect. Upon successful duplication, a new Tumor Cell with inherited DNA is placed in a neighbouring empty location. Beyond its stepwise behaviour, the Tumor Cell contributes to angiogenesis by exposing an Angiogenesis Effect, may present tumor antigens depending on its DNA configuration, and it can inhibit Cytotoxic T Cell activity through PD1 pathway activation unless *ICI* therapy is active.


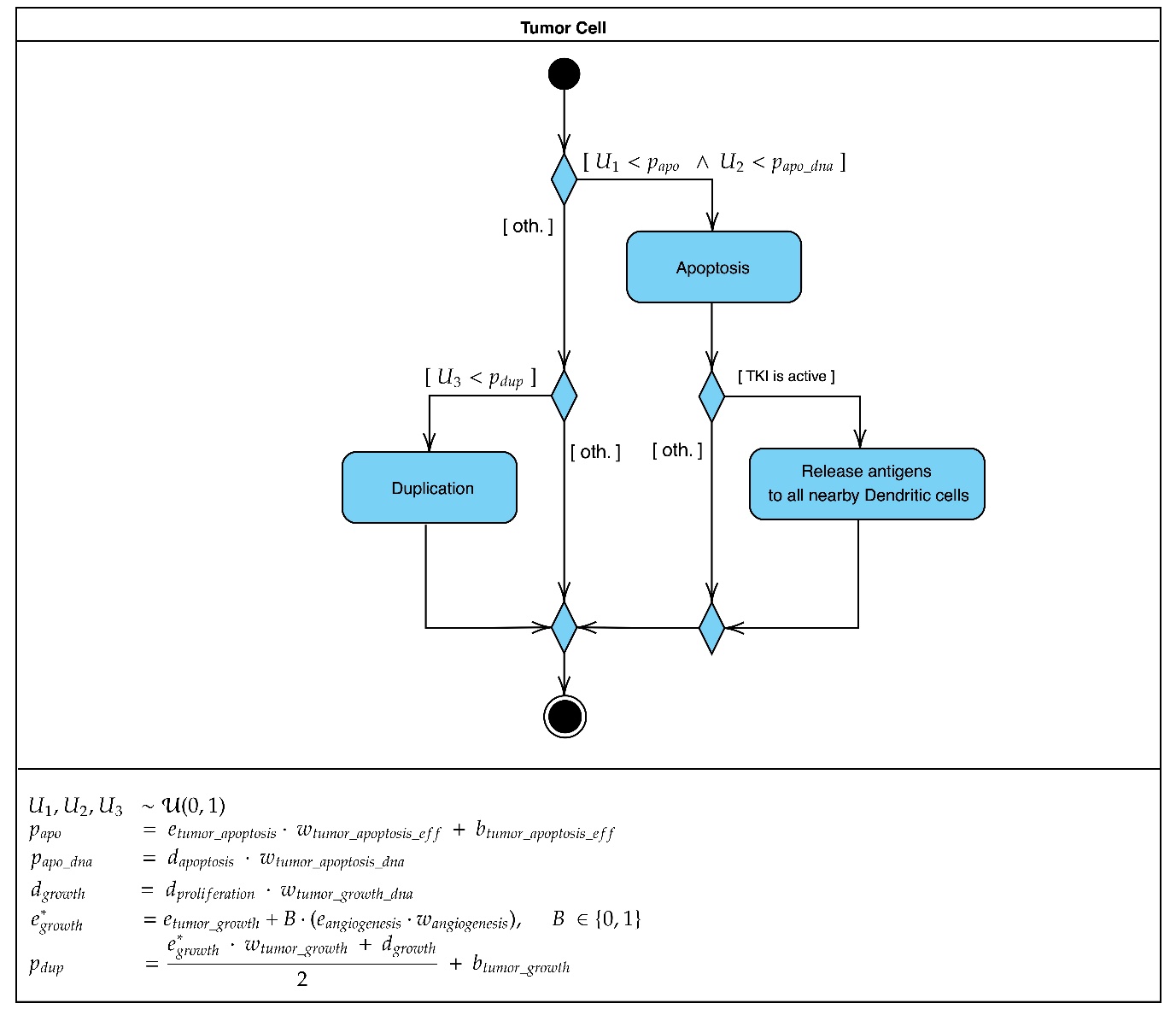


**FIGURE S6.** Behavior of a Cytotoxic T Cell at each time step of the simulation. At every step, the Cytotoxic T Cell first evaluates apoptosis, which depends on the experienced T Cell Apoptosis Effect and a baseline apoptosis bias. If apoptosis is triggered, the cell is removed from the model. If the cell survives, it undergoes sex hormone stimulation, which modulates its Kill Rate Effect, proliferation probability, and apoptosis susceptibility according to local levels of Estrogen, Progesterone, and Testosterone. The Cytotoxic T Cell then evaluates proliferation, with probability depending on its intrinsic proliferation rate, activation level, and model weights. The cell subsequently moves towards nearby Tumor Cells within a weighted search radius. If a Tumor Cell is found, the Cytotoxic T Cell attempts to kill it with a probability determined by its Kill Rate Effect and activation level. Successful killing induces progressive exhaustion, reducing activation. If the Tumor Cell activates PD1-mediated inhibition, the Cytotoxic T Cell instead experiences a reduction in activation proportional to checkpoint pathway strength.


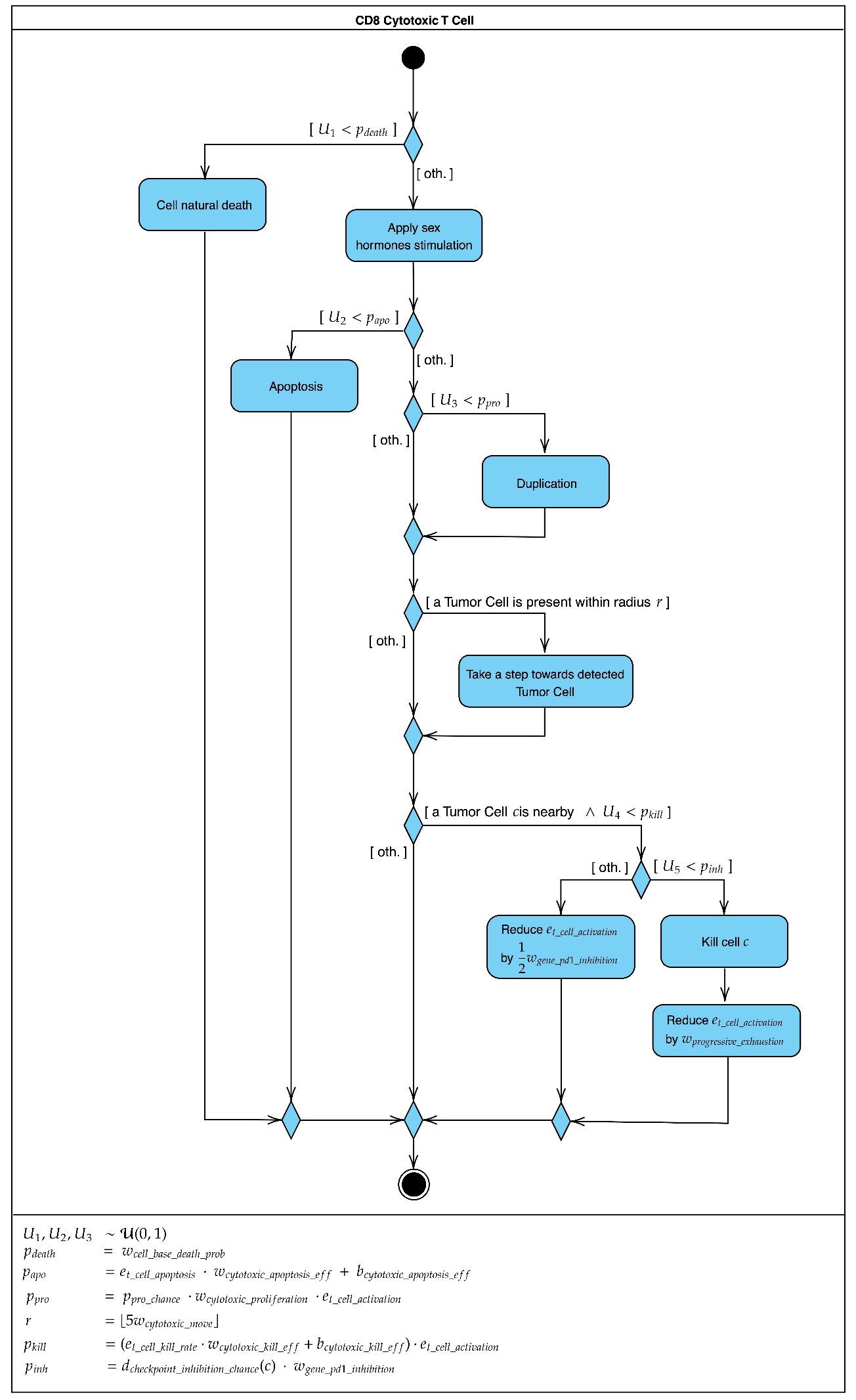


**FIGURE S7.** Behavior of a CD4 Naive T Cell at each time step of the simulation. Initially, the naive CD4 cell applies a hormonal decay effect which reduces the influence of surrounding hormones over time - it mimics surrounding hormone depletion. Then it perceives the surroundings for new sex hormones. Stimulation levels are then calculated from the perception. If it applies, the cell differentiates into T regulatory cell, otherwise the step comes to an end.


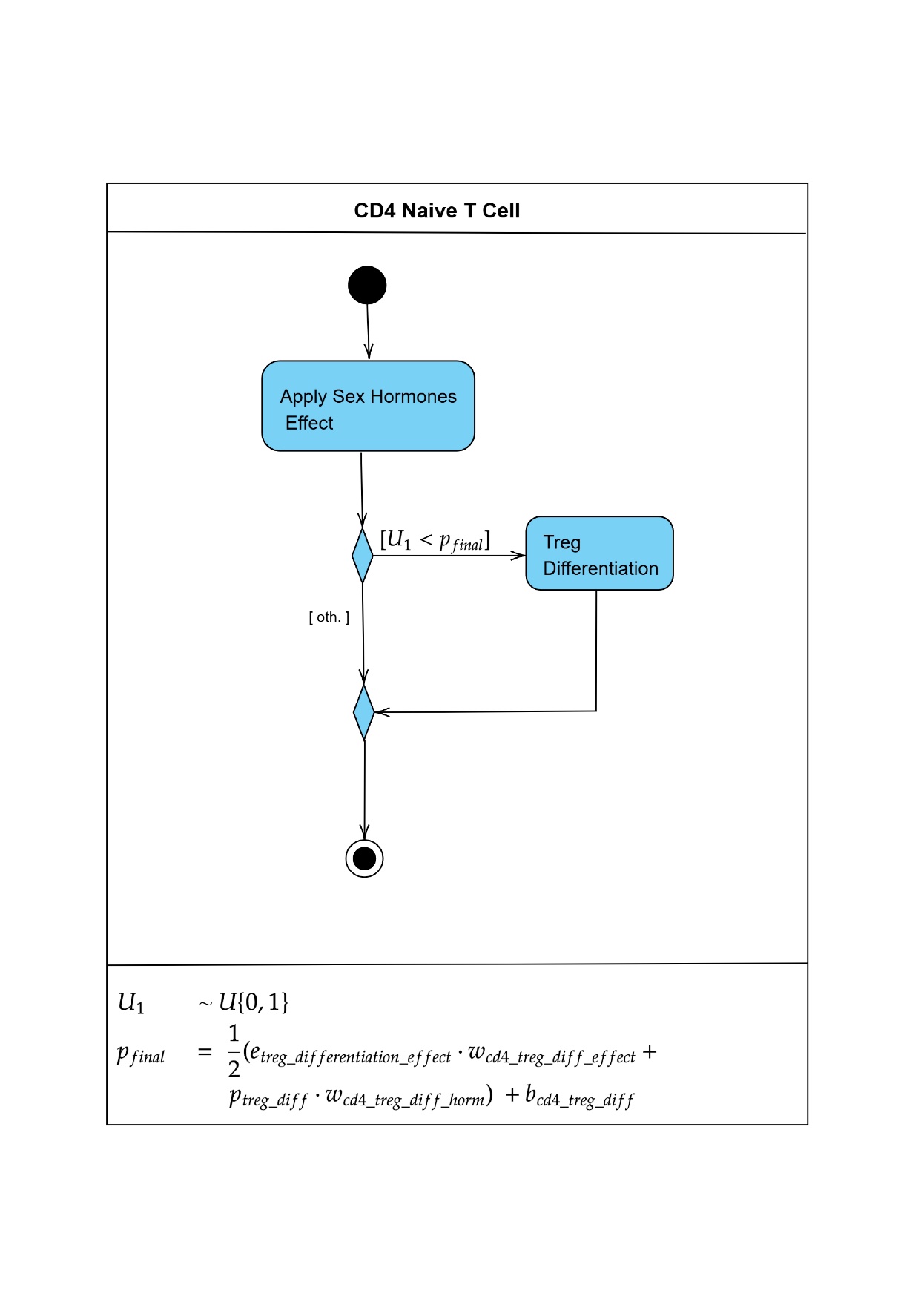


**FIGURE S8.** The figure illustrates the behavior of the CD4+ Helper 1 T cell. It starts by checking if the cell has reached its natural lifespan, in that case its activities in the simulation would come to an end and it would be removed from the environment. The cell proceeds to perceive the sex hormones within a given radius to determine their quantity and thereby how much influence they bring to it. When possible, the cell will activate M1 macrophages and dendritic cells, as well as undergo duplication. The cell actively moves towards tumor cells, specifically, the closest one. Finally, the immune infiltration factor, which represents the number of cells that can be called into action by this cell, is reset to the default value, compensating for the instance where it might have been modified during treatment.


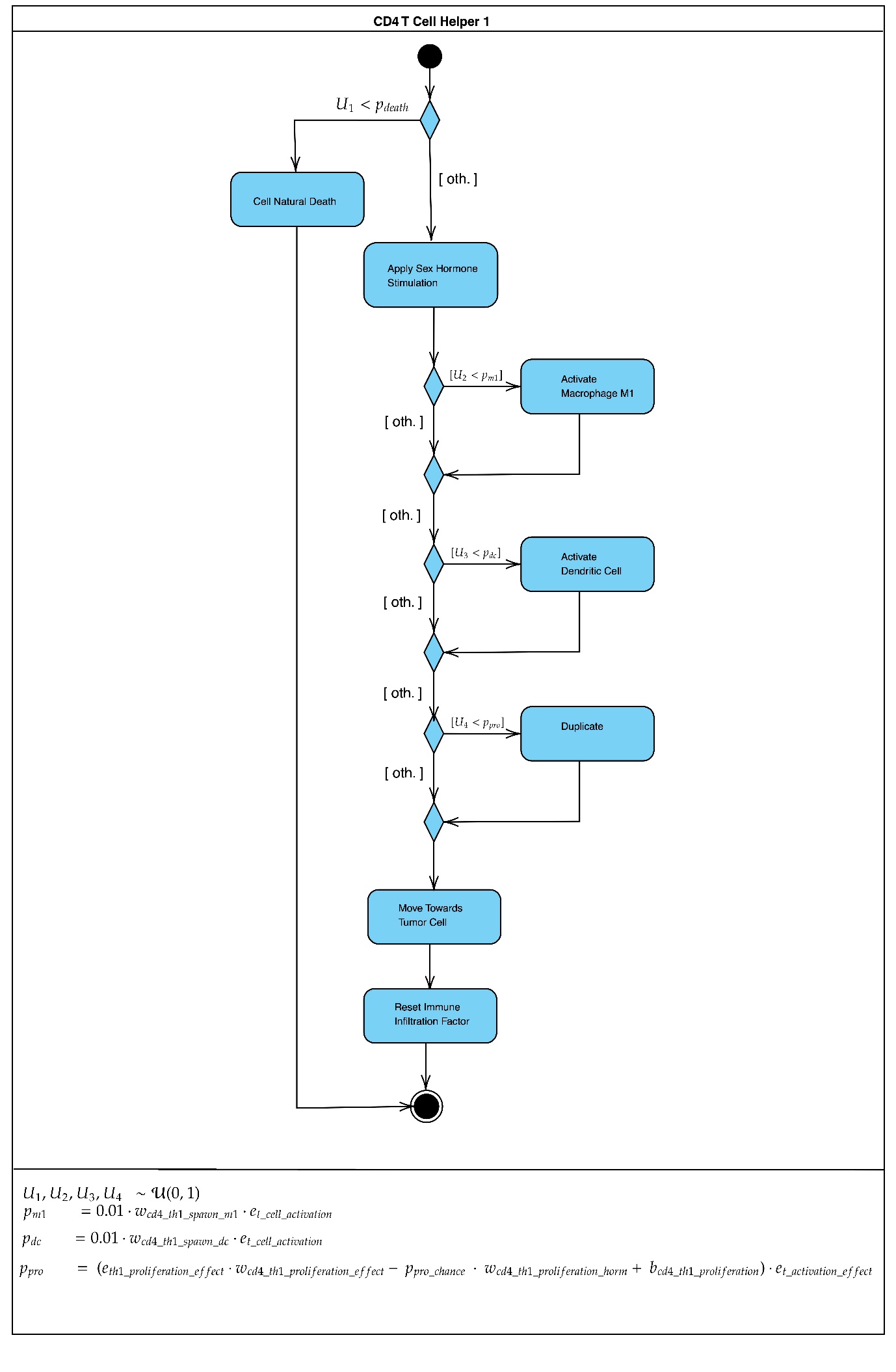


**FIGURE S9.** The CD4+ Helper 2 T cell starts by checking if the cell has reached its natural lifespan. Be that the case, its activities in the simulation would cease, and it would be removed from the environment. Otherwise, the cell proceeds to perceive the sex hormones within a given radius to determine their quantity and thereby how much influence they bring to it. When possible, the cell will activate M1 macrophages and attract CD8+ T cells, as well as undergo duplication. The cell actively moves towards tumor cells, specifically, the closest one within its search dimension. Finally, the immune infiltration factor, which represents the number of cells that can be called into action by this cell, is reset to the default value, compensating for the instance where it might have been modified during treatment.


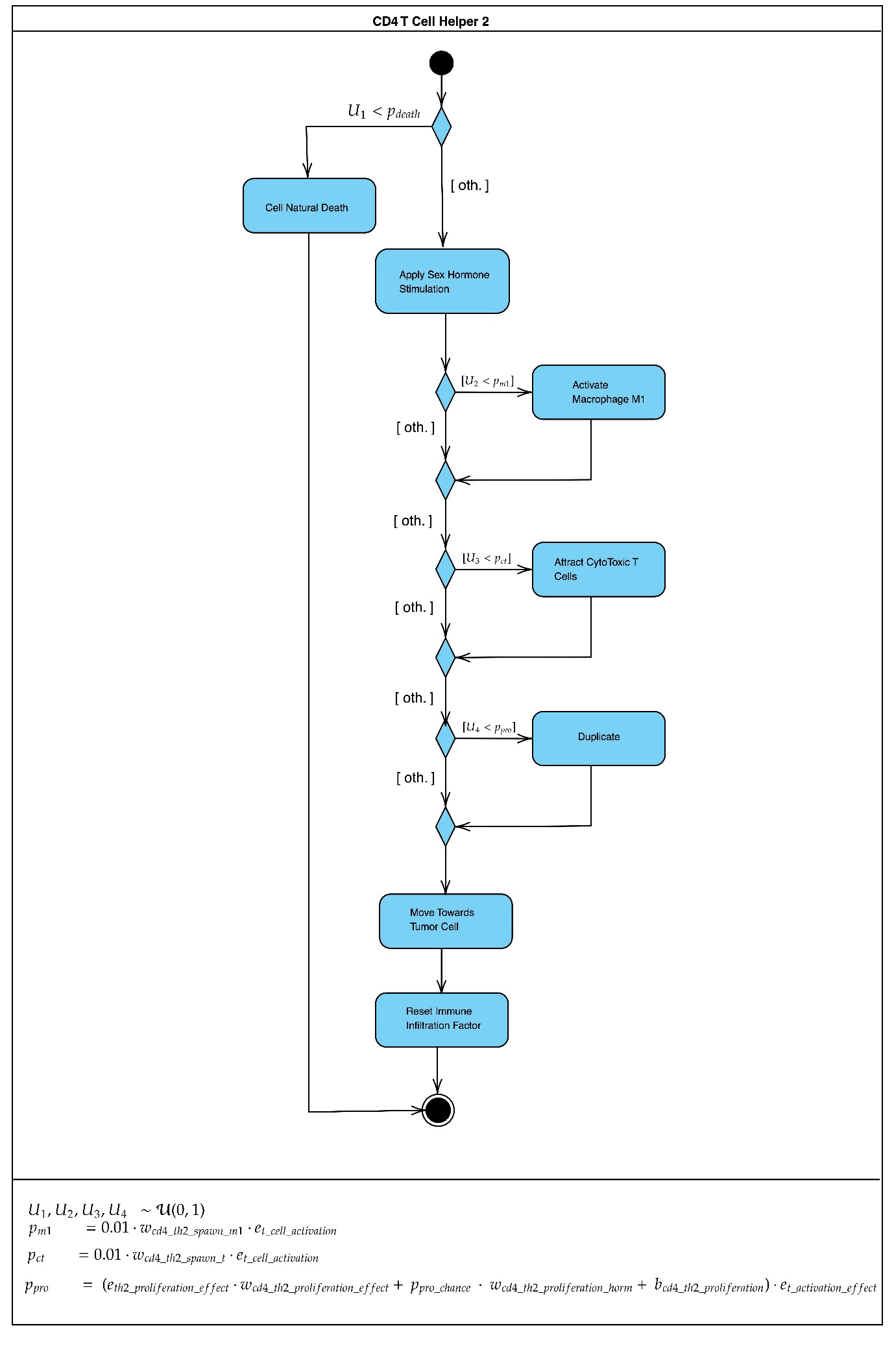


**TABLE LEGEND**

**TABLE S1.** Baseline immune cell concentrations and deviation step values used for synthetic data generation. Each baseline value represents the mean value tailored to the model as defined in OncoAgent. Each step value corresponds to a fixed additive proportional deviation from the baseline value, which is applied according to the step conversion table.

| CELL TYPE | BASELINE (cells/mL) | DEV. PER '+' OR '-' |
| --- | --- | --- |
| *Cytotoxic T Cells* | 80,000 | ± 8,000 |
| *Treg Cells* | 40,000 | ± 4,000 |
| *Mast Cells* | 40,000 | ± 4,000 |
| *PDC* | 40,000 | ± 4,000 |
| *Th1 Cells* | 40,000 | ± 4,000 |
| *Th2 Cells* | 40,000 | ± 4,000 |
| *Dendritic Cells* | 80,000 | ± 8,000 |
| *M1 Macrophages* | 40,000 | ± 4,000 |
| *M2 Macrophages* | 40,000 | ± 4,000 |
| *NKL Cells* | 160,000 | ± 16,000 |
| *CD4 T Cells* | 80,000 | ± 8,000 |
| *CD8 T Cells* | 80,000 | ± 8,000 |
| *Neutrophils* | 160,000 | ± 16,000 |

**TABLE S2.** Complete list of learnable parameters optimized in the reported ranges.

| Parameter | Type | Default | Learnable Range | Description |
| --- | --- | --- | --- | --- |
| *w_BMI_on_treg_diff_* | float | 0.01 | [0.25,4] | Influence of BMI on Treg differentiation |
| *w_BMI_on_m1_mutation_* | float | 0.01 | [0.25,4] | Influence of BMI on M1 macrophage mutation |
| *w_BMI_on_m2_mutation_* | float | 0.01 | [0.25,4] | Influence of BMI on M2 macrophage mutation |
| *w_BMI_nkl_kill_rate_* | float | 0.01 | [0.25,4] | Influence of BMI on NKL kill rate |
| *w_treg_move_* | float | 1 | [0.25,4] | Influence of Treg move radius on movement |
| *w_cytotoxic_move_* | float | 1 | [0.25,4] | Influence of cytotoxic T cell look-up size for tumors |
| *w_m1_mutation_* | float | 1 | [0.25,4] | Threshold for M1 macrophage mutation chance |
| *w_m1_move_* | float | 1 | [0.25,4] | Influence of M1 macrophage look-up size for movement |
| *w_m1_phagocytosis_* | float | 1 | [0.25,4] | Influence of M1 macrophage phagocytosis chance |
| *w_m1_digest_* | float | 1 | [0.25,4] | Influence of M1 macrophage digestion rate |
| *w_m1_t_kill_rate_* | float | 1 | [0.25,4] | Influence of M1 macrophage on T-kill rate |
| *w_m1_th1_proliferation_* | float | 1 | [0.25,4] | Influence of M1 macrophages on Th1 proliferation |
| *w_th1_proliferation_* | float | 1 | [0.25,4] | Influence on Th1 proliferation |
| *w_dc_phagocytosis_eff_* | float | 1 | [0.25,4] | Influence of dendritic cell phagocytosis effect |
| *w_m2_mutation_* | float | 1 | [0.25,4] | Threshold for M2 macrophage mutation chance |
| *w_m2_move_* | float | 1 | [0.25,4] | Influence of M2 macrophage look-up size for movement |
| *w_m2_t_kill_rate_* | float | 1 | [0.25,4] | Influence of M2 macrophages on T-kill rate |
| *w_m2_tumour_growth_* | float | 1 | [0.25,4] | Influence of M2 macrophages on tumour growth |
| *w_m2_angiogenesis_* | float | 1 | [0.25,4] | Influence of M2 macrophages on angiogenesis |
| *w_sex_hormone_cd8_* | float | 1 | [0.25,4] | Global influence of hormones on CD8+ cells |
| *w_tumor_apoptosis_eff_* | float | 1 | [0.25,4] | Influence of effects on tumor apoptosis threshold |
| *w_tumor_apoptosis_dna_* | float | 1 | [0.25,4] | Influence of mutations on tumor apoptosis threshold |
| *w_tumor_growth_eff_* | float | 0.6 | [0.25,4] | Influence of effects on tumor growth threshold |
| *w_tumor_growth_dna_* | float | 1 | [0.25,4] | Influence of mutations on tumor growth threshold |
| *w_tumor_angiogenesis_* | float | 1 | [0.25,4] | Influence of effects on tumor angiogenesis threshold |
| *w_antigen_presentation_* | float | 1 | [0.25,4] | Influence of antigen presentation mutations |
| *w_angiogenesis_tumor_growth_* | float | 1 | [0.25,4] | Influence of angiogenesis on tumor growth chance |
| *w_gene_pd1_inhibition_* | float | 1 | [0.25,4] | Influence of gene mutations on PD-1 inhibition |
| *w_cd4_treg_diff_eff_* | float | 1 | [0.25,4] | Influence of effects on Treg differentiation threshold |
| *w_cd4_treg_diff_horm_* | float | 1 | [0.25,4] | Influence of hormones on Treg differentiation threshold |
| *w_cd4_th1_proliferation_eff_* | float | 1 | [0.25,4] | Influence of effects on Th1 proliferation threshold |
| *w_cd4_th1_proliferation_horm_* | float | 1 | [0.25,4] | Influence of hormones on Th1 proliferation threshold |
| *w_cd4_th1_spawn_m1_* | float | 1 | [0.25,4] | Influence of Th1 on M1 macrophage spawn chance |
| *w_cd4_th1_spawn_dc_* | float | 1 | [0.25,4] | Influence of Th1 on dendritic cell spawn chance |
| *w_cd4_th2_spawn_m1_* | float | 1 | [0.25,4] | Influence of Th2 on M1 macrophage spawn chance |
| *w_cd4_th2_spawn_t_* | float | 1 | [0.25,4] | Influence of Th2 on cytotoxic T cell spawn chance |
| *w_cd4_th2_proliferation_eff_* | float | 1 | [0.25,4] | Influence of effects on Th2 proliferation threshold |
| *w_cd4_th2_proliferation_horm_* | float | 1 | [0.25,4] | Influence of hormones on Th2 proliferation threshold |
| *w_cytotoxic_proliferation_* | float | 1 | [0.25,4] | Influence of effects on cytotoxic T cell proliferation threshold |
| *w_cytotoxic_apoptosis_* | float | 1 | [0.25,4] | Influence of effects on cytotoxic T cell apoptosis threshold |
| *w_cytotoxic_kill_* | float | 1 | [0.25,4] | Influence of effects on cytotoxic T cell kill threshold |
| *w_cytotoxic_pd1_inhibition_* | float | 1 | [0.25,4] | Influence of PD-1 inhibition on cytotoxic T cell behavior |
| *w_mast_cell_angiogenesis_* | float | 1 | [0.25,4] | Influence of mast cells on angiogenesis chance |
| *w_mast_cell_m1_mutation_* | float | 1 | [0.25,4] | Influence of mast cells on M1 mutation chance |
| *w_mast_cell_t_kill_rate_* | float | 1 | [0.25,4] | Influence of mast cells on T-kill rate chance |
| *w_mast_cell_tumour_apoptosis_* | float | 1 | [0.25,4] | Influence of mast cells on tumor apoptosis chance |
| *w_mast_cell_tumour_growth_* | float | 1 | [0.25,4] | Influence of mast cells on tumor growth chance |
| *w_mast_cell_spawn_dc_* | float | 1 | [0.25,4] | Influence of mast cells on dendritic cell spawn chance |
| *w_natural_killer_kill_rate_* | float | 1 | [0.25,4] | Influence of kill rate effect on natural killer cells |
| *w_nkl_t_kill_rate_* | float | 1 | [0.25,4] | Influence of NKL on T-kill rate chance |
| *w_pdc_nkl_spawn_* | float | 1 | [0.25,4] | Influence of plasmacytoid dendritic cells on NKL spawn chance |
| *w_pdc_angiogenesis_* | float | 1 | [0.25,4] | Influence of plasmacytoid dendritic cells on angiogenesis chance |
| *w_pdc_treg_diff_* | float | 1 | [0.25,4] | Influence of plasmacytoid dendritic cells on Treg differentiation chance |
| *w_pdc_t_proliferation_* | float | 1 | [0.25,4] | Influence of plasmacytoid dendritic cells on T cell proliferation chance |
| *w_pdc_t_kill_* | float | 1 | [0.25,4] | Influence of plasmacytoid dendritic cells on T cell kill chance |
| *w_pdc_nkl_kill_* | float | 1 | [0.25,4] | Influence of plasmacytoid dendritic cells on NKL kill chance |
| *w_treg_t_kill_rate_* | float | 1 | [0.25,4] | Influence of Treg on T-kill rate chance |
| *w_treg_t_proliferation_* | float | 1 | [0.25,4] | Influence of Treg on T cell proliferation chance |
| *w_treg_t_apoptosis_* | float | 1 | [0.25,4] | Influence of Treg on T cell apoptosis chance |
| *w_treg_activation_* | float | 1 | [0.25,4] | Influence of Treg on T cell activation chance |
| *w_treg_dc_phagocytosis_* | float | 1 | [0.25,4] | Influence of Treg on dendritic cell phagocytosis chance |
| *w_search_dimension_* | float | 0.5 | [0.25,4] | Search dimension for agents |
| *b_m1_mutation_* | float | 0 | [-1,1] | Bias adjusting the base mutation chance for M1 macrophages |
| *b_th1_proliferation_* | float | 0 | [-1,1] | Bias adjusting the base effect influence on Th1 proliferation |
| *b_dc_phagocytosis_* | float | 0 | [-1,1] | Bias adjusting the base probability of dendritic cell phagocytosis |
| *b_tumor_apoptosis_* | float | 0 | [-1,1] | Bias adjusting the base threshold for tumor apoptosis chance |
| *b_tumor_growth_* | float | 0 | [-1,1] | Bias adjusting the base threshold for tumor growth chance |
| *b_tumor_angiogenesis_* | float | 0 | [-1,1] | Bias adjusting the base threshold for tumor angiogenesis chance |
| *b_antigen_presentation_* | float | 0 | [-1,1] | Bias adjusting the base antigen presentation chance for tumor cells |
| *w_tumour_growth_threshold_* | float | 1 | [0.75,0.99] | Weight adjusting the terminal condition for tumor growth threshold |
| *receptor_threshold_variation* | int | 1 | [0,3] | Variation in receptor threshold for T cells |
| *w_ici_effectiveness_* | float | 1 | [0,1] | Weight adjusting the effectiveness of ICI drug |
| *w_tki_effectiveness_* | float | 1 | [0,1] | Weight adjusting the effectiveness of TKI drug |
| *w_cell_base_death_prob_* | float | 0.009 | [0.001,0.1] | Probability of natural cell death |
| *w_progressive_exhaustion_* | float | 0.05 | [0.005,0.3] | Exhaustion impact of successful kills in T cells |

**TABLE S3.** Impact of learnable parameters on error during training. The table is split by optimization function, listing the top five parameters ranked by absolute correlation with error.

| **Concordance Index (*CI*)** | | **Mean Square Error (*MSE*)** | |
| --- | --- | --- | --- |
| **PARAMETER** | **CORRELATION WITH ERROR** | **PARAMETER** | **CORRELATION WITH ERROR** |
| $w_{cd4\_th2\_spawn\_t}$ | 0.767313 | $w_{tumor\_apoptosis\_dna}$ | 0.536786 |
| $w_{search\_dimension}$ | 0.674132 | $w_{BMI on\_m1\_mutation}$ | 0.470902 |
| $w_{pdc\_t\_proliferation}$ | 0.670114 | $w_{pdc\_nkl\_spawn}$ | 0.465960 |
| $b_{natural\_killer\_kill\_rate}$ | -0.626136 | $w_{BMI\_nkl\_kill\_rate}$ | -0.406357 |
| $w_{ici\_effectiveness}$ | 0.624777 | $b_{antigen\_presentation}$ | 0.403357 |

**TABLE S4.** Patient and simulation-specific parameters used to initialize the model.

| MODEL PARAMETERS | | | |
| --- | --- | --- | --- |
| Parameter | **Type** | **Default** | **Description** |
| *volume* | float | 0.0001 | Volume (mL) |
| *block_size* | int | 10 | Block Size (μm) |
| *random_seed* | int | 1 | Random Seed |
| *max_steps* | int | 500 | Maximum number of simulation steps |
| PATIENT PARAMETERS | | | |
| Parameter | Type | Default | Description |
| *sex* | str | F | Sex (F/M) |
| *BMI* | float | 22.0 | Body Mass Index (kg/m²) |
| *treatment* | str | ICI+TKI | Drugs used in Treatment (ICI/TKI) |
| *treatment_start* | int | 100 | Treatment Start (steps) |
| *ctc_concentration* | int | 80000 | CTC Concentration (cells/mL) |
| *neutrophil_concentration* | int | 40000 | Neutrophil Concentration (cells/mL) |
| *mast_cell_concentration* | int | 40000 | Mast Cell Concentration (cells/mL) |
| *treg_concentration* | int | 40000 | Treg Cell Concentration (cells/mL) |
| *pdc_concentration* | int | 40000 | PDC Concentration (cells/mL) |
| *th1_concentration* | int | 40000 | Th1 Cell Concentration (cells/mL) |
| *th2_concentration* | int | 40000 | Th2 Cell Concentration (cells/mL) |
| *dc_concentration* | int | 80000 | DC Concentration (cells/mL) |
| *m1_concentration* | int | 40000 | M1 Macrophage Concentration (cells/mL) |
| *m2_concentration* | int | 40000 | M2 Macrophage Concentration (cells/mL) |
| *nkl_concentration* | int | 160000 | NKL Cell Concentration (cells/mL) |
| *cd4_concentration* | int | 80000 | CD4 T Cell Concentration (cells/mL) |
| *cd8_concentration* | int | 80000 | CD8 T Cell Concentration (cells/mL) |
| *NKL_delta* | int | 16000 | NK Cells Difference |

**TABLE S5.** Scoring system table describing predictive influence of clinical features on TME immune cell concentrations in RCC. Symbols indicate direction of association: ++ strong positive; + positive; - negative; 0 no consistent association.

| Clinical Feature | CTL | Treg | Mast | pDC | Th1 | Th2 | DC | M1 | M2 | NK | CD4 | CD8 | TME Neut |
| --- | --- | --- | --- | --- | --- | --- | --- | --- | --- | --- | --- | --- | --- |
| *Female sex* | **+** | **+** | **0** | **+** | **+** | **0** | **+** | **+** | **0** | **0** | **+** | **+** | **0** |
| *Male sex* | **0** | **0** | **0** | **0** | **0** | **+** | **0** | **0** | **+** | **+** | **0** | **0** | **+** |
| *Smoking* | **-** | **+** | **+** | **-** | **-** | **+** | **-** | **-** | **+** | **-** | **-** | **-** | **+** |
| *High BMI* | **-** | **+** | **+** | **-** | **-** | **+** | **-** | **-** | **+** | **-** | **-** | **-** | **+** |
| *Sarcomatoid differentiation* | **++** | **-** | **+** | **0** | **+** | **-** | **+** | **++** | **-** | **+** | **+** | **++** | **-** |
| *Clear cell histology* | **+** | **0** | **+** | **0** | **+** | **0** | **+** | **+** | **-** | **+** | **+** | **+** | **-** |
| *Chromophobe RCC* | **-** | **-** | **-** | **0** | **-** | **-** | **-** | **-** | **+** | **-** | **-** | **-** | **-** |
| *Papillary RCC* | **-** | **+** | **+** | **-** | **-** | **+** | **-** | **-** | **+** | **-** | **-** | **-** | **+** |
| *High NLR* | **-** | **+** | **+** | **-** | **-** | **+** | **-** | **-** | **+** | **-** | **-** | **-** | **++** |
| *High Platelet count* | **-** | **+** | **+** | **-** | **-** | **+** | **-** | **-** | **+** | **-** | **-** | **-** | **+** |
| *ECOG >1* | **-** | **+** | **+** | **-** | **-** | **+** | **-** | **-** | **+** | **-** | **-** | **-** | **+** |
| *Lung metastases* | **+** | **-** | **+** | **0** | **+** | **-** | **+** | **+** | **-** | **+** | **+** | **+** | **-** |
| *Bone metastases* | **-** | **+** | **+** | **-** | **-** | **+** | **-** | **-** | **+** | **-** | **-** | **-** | **+** |
| *IMDC poor risk* | **-** | **+** | **+** | **-** | **-** | **+** | **-** | **-** | **+** | **-** | **-** | **-** | **+** |
| *G3-G4 hypothyroidism (irAE)* | **++** | **-** | **-** | **+** | **+** | **-** | **+** | **+** | **-** | **+** | **+** | **+** | **-** |

**TABLE S6.** Training data obtained from the selected subset of patients in the ARON dataset. The first row below the column headers illustrates the conversion rules used. Specifically, the treatment type is derived from the reported drugs in the first-line immunotherapy, while the “Start” and “OS” values are calculated by mapping 100 simulation steps to one year.

| Patient | ID | Treatment | Start | BMI | Sex | CTC | Treg | Mast | PDC | Th1 | Th2 | DC | M1 | M2 | NKL | CD4 | CD8 | Neut | OS | Death |
| --- | --- | --- | --- | --- | --- | --- | --- | --- | --- | --- | --- | --- | --- | --- | --- | --- | --- | --- | --- | --- |
|  | **Patient code** | **First-line immune-combo therapy** | **(Start date – Met. Date) / 365** | **Weight / Height^2^** | **Sex  (1→M, 2→ F)** | **See tables S1 and S5** | | | | | | | | | | | | | **(Death/Last FU − Met. Date) / 365** | **Death (0→F, 1→ T)** |
| *p1* | ***62*** | ***ICI*** | ***30*** | ***21.97*** | ***M*** | ***64000*** | ***44000*** | ***48000*** | ***28000*** | ***32000*** | ***52000*** | ***64000*** | ***32000*** | ***48000*** | ***160000*** | ***64000*** | ***64000*** | ***192000*** | ***331*** | ***T*** |
| *p2* | ***99*** | ***ICI*** | ***15*** | ***22.46*** | ***M*** | ***72000*** | ***40000*** | ***44000*** | ***32000*** | ***36000*** | ***48000*** | ***72000*** | ***36000*** | ***44000*** | ***176000*** | ***72000*** | ***64000*** | ***192000*** | ***80*** | ***T*** |
| *p3* | ***124*** | ***ICI*** | ***29*** | ***27.74*** | ***M*** | ***64000*** | ***44000*** | ***48000*** | ***28000*** | ***32000*** | ***52000*** | ***64000*** | ***32000*** | ***48000*** | ***160000*** | ***64000*** | ***56000*** | ***208000*** | ***387*** | ***F*** |
| *p4* | ***59*** | ***ICI+TKI*** | ***119*** | ***26.56*** | ***F*** | ***80000*** | ***52000*** | ***60000*** | ***32000*** | ***40000*** | ***48000*** | ***80000*** | ***40000*** | ***48000*** | ***144000*** | ***80000*** | ***80000*** | ***192000*** | ***205*** | ***T*** |
| *p5* | ***34*** | ***ICI+TKI*** | ***41*** | ***21.97*** | ***F*** | ***72000*** | ***56000*** | ***56000*** | ***32000*** | ***36000*** | ***52000*** | ***72000*** | ***36000*** | ***48000*** | ***128000*** | ***72000*** | ***72000*** | ***208000*** | ***76*** | ***T*** |
| *p6* | ***40*** | ***ICI+TKI*** | ***3*** | ***24.34*** | ***F*** | ***72000*** | ***56000*** | ***56000*** | ***32000*** | ***36000*** | ***52000*** | ***72000*** | ***36000*** | ***44000*** | ***144000*** | ***72000*** | ***72000*** | ***224000*** | ***129*** | ***F*** |
| *p7* | ***44*** | ***ICI+TKI*** | ***28*** | ***25.54*** | ***F*** | ***68000*** | ***68000*** | ***70400*** | ***28000*** | ***68000*** | ***70400*** | ***65600*** | ***68000*** | ***70400*** | ***144000*** | ***65600*** | ***65600*** | ***70400*** | ***107*** | ***F*** |

**TABLE S7.** Sex-dependent spawning probabilities of hormones in the tumor microenvironment at each simulation step.

| **SEX** | **ESTROGEN** | **PROGESTERONE** | **TESTOSTERONE** |
| --- | --- | --- | --- |
| *Male (M)* | 20% | 10% | 100% |
| *Female (F)* | 90% | 80% | 20% |

**TABLE S8.** Modeled influence of sex hormones on T cells functional properties. E, T and P represent exponentially decayed cumulative perception of nearby estrogens, testosterone, and progesterone hormones respectively. Positive signs indicate stimulation; negative signs indicate inhibition. All effects on CD8 Cytotoxic T Cells are scaled by the learnable parameter $w_{h} = w_{sex\_hormone\_CD8}$ , while $\sigma$ indicates the sigmoid function.

| CELL | KILL RATE EFFECT | PROLIFERATION CHANCE | APOPTOSIS EFFECT | DIFFERENTIATION CHANCE |
| --- | --- | --- | --- | --- |
| *CD8 Cytotoxic T Cell* | $+0.1 w_{h}E -0.1 w_{h}T$ | $0.015 +0.005 w_{h}E -0.005 w_{h}T -0.005 w_{h}P$ | $- 0.005 w_{h}E +0.005 w_{h}T +0.005 w_{h}P$ | *---* |
| *CD4 T Cell* | *---* | *---* | *---* | $\sigma(-6.0 + 1.2 E + 1.0 P + 0.4 T)$ |
| *CD4 T Helper 1 Cell* | *---* | $\sigma(-6.0 + 1.2 E + 1.0 P + 0.4 T)$ | *---* | *---* |
| *CD4 T Helper 2 Cell* | *---* | $\sigma(-6.0 + 1.2 E + 1.0 P + 0.4 T)$ | *---* | *---* |

**TABLE S9.** Lists of drug-induced effects on the simulated TME when applied in proportion and learnable weight $p, w \in\left[ 0, 1 \right]$, respectively, resulting in effectiveness $f=p\cdot w$. The descriptions correspond directly to the implementation rules within the model.

| **DRUG CLASS** | **PER-STEP EFFECT DESCRIPTION WITH EFFECTIVENESS** $f$ |
| --- | --- |
| *ICI* | - *Sets to 0 the probability of* $f$ *% of all tumor cells to present the PD-1/PD-L1 inhibitor.* - *Restores T cell activity of* $f$*% of all T cells in the tumor microenvironment (TME).* - *Increases immune infiltration, so doubles the* $f$*% of all immune cells that get to the TME when called.* |
| *TKI* | - *Reduces reproduction probability of* $f$*% of all tumor cells by multiplying their* $e_{tumor growth}$ *by* $1-f$*.* - *Inhibits pro-tumoral influence of angiogenesis by multiplying the experienced* $e_{angiogenesis}$*of* $f$*% of all tumor cells by* $1-f$*.* - *Promotes antigen release by letting* $f$*% of all tumor cells be set to release neoantigens when dying.* - $f$*% of T Cells reduce the probability of turning into Treg by multiplying their* $e_{t reg differentiation}$ *by* $1-f$*.* |

**ALGORITHM S1.** High-level pseudocode of the agent-based evolutionary process implementing the Genetic Algorithm. In our model, $S$ represents the state transition function induced by the running simulation, $n = 10$, $m = 3$ and the termination predicate $T$ is given by:

$$T\left( s \right)= \left\{ \begin{aligned} 1 \mathrm{if} t\left( s \right)\geq T_{\max} \\ 1 \mathrm{if} t\left( s \right)<T_{\max}\wedge N\left( s \right)=0 \\ \begin{matrix} 1 \mathrm{if} t\left( s \right)<T_{\max}\wedge N\left( s \right)\geq N_{\max} \\ 1 \mathrm{if} t\left( s \right)<T_{\max}\wedge0<N\left( s \right)<N_{\max}\wedge g\left( s \right)\geq w_{tumor\_growth\_threshold} \\ 0 otherwise \end{matrix} \end{aligned} \right.$$

Where:

- $t\left( s \right)$ denotes the current simulation step,
- $N\left( s \right)$ is the number of tumor cells in state $s$,,
- $T_{max}$ is the maximum number of simulation steps,
- $N_{max}$ is the maximum number of tumor cells,
- $g\left( s \right)$ is the average tumor growth rate that, given $g_{c}$ the growth-rate value of cell $c$ , is defined as:

$$g\left( s \right)= \left\{ \begin{aligned} \frac{1}{N(s)} \sum_{c\in P(s)} g_{c} \mathrm{if} N\left( s \right)>0 \\ 0 \mathrm{if} N\left( s \right)=0 \end{aligned} \right.$$

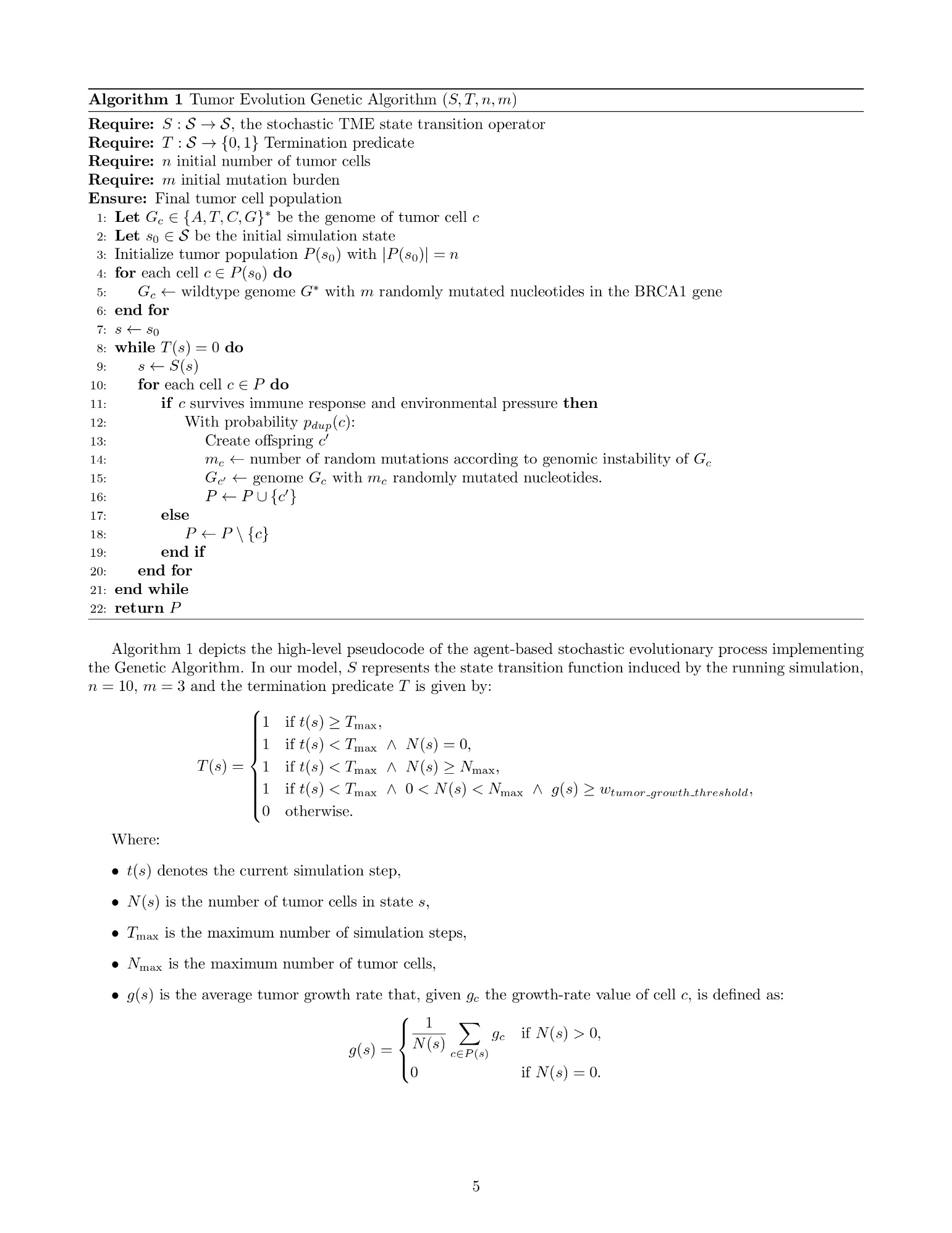

Supplement: Supplementary file 1 [file DataSheet1.docx]
